# The RNA helicase Dbp7 promotes domain V/VI compaction and stabilization of inter-domain interactions during early 60S assembly

**DOI:** 10.1038/s41467-021-26208-9

**Published:** 2021-10-22

**Authors:** Gerald Ryan R. Aquino, Philipp Hackert, Nicolai Krogh, Kuan-Ting Pan, Mariam Jaafar, Anthony K. Henras, Henrik Nielsen, Henning Urlaub, Katherine E. Bohnsack, Markus T. Bohnsack

**Affiliations:** 1 Department of Molecular Biology, University Medical Center Göttingen, Humboldtallee 23, 37073 Göttingen, Germany; 2 Department of Cellular and Molecular Medicine, University of Copenhagen, 2200N Copenhagen, Denmark; 3 Max Planck Institute for Biophysical Chemistry, Bioanalytical Mass Spectrometry, 37077 Göttingen, Germany; 4 Molecular, Cellular and Developmental Biology Unit (MCD), Centre de Biologie Intégrative (CBI), Université de Toulouse, CNRS, UPS, 31062 Toulouse, France; 5 Genomics Group, Faculty of Biosciences and Aquaculture, Nord University, 8049 Bodø, Norway; 6 Institute for Clinical Chemistry, University Medical Center Göttingen, 37075 Göttingen, Germany; 7 Göttingen Centre for Molecular Biosciences, Georg-August-University, Justus-von-Liebig Weg 11, 37077 Göttingen, Germany; 8 Present Address: Hematology/Oncology, Department of Medicine II, Johann Wolfgang Goethe University, 60590 Frankfurt am Main, Germany; 9 Present Address: Frankfurt Cancer Institute, Goethe University, 60596 Frankfurt am Main, Germany

**Keywords:** Enzyme mechanisms, RNA folding

## Abstract

Early pre-60S ribosomal particles are poorly characterized, highly dynamic complexes that undergo extensive rRNA folding and compaction concomitant with assembly of ribosomal proteins and exchange of assembly factors. Pre-60S particles contain numerous RNA helicases, which are likely regulators of accurate and efficient formation of appropriate rRNA structures. Here we reveal binding of the RNA helicase Dbp7 to domain V/VI of early pre-60S particles in yeast and show that in the absence of this protein, dissociation of the Npa1 scaffolding complex, release of the snR190 folding chaperone, recruitment of the A3 cluster factors and binding of the ribosomal protein uL3 are impaired. uL3 is critical for formation of the peptidyltransferase center (PTC) and is responsible for stabilizing interactions between the 5′ and 3′ ends of the 25S, an essential pre-requisite for subsequent pre-60S maturation events. Highlighting the importance of pre-ribosome remodeling by Dbp7, our data suggest that in the absence of Dbp7 or its catalytic activity, early pre-ribosomal particles are targeted for degradation.

## Abstract (web summary)

Early steps of large 60S ribosomal subunit biogenesis are not well understood. Here, the authors combine biochemical experiments with protein-RNA crosslinking and mass spectrometry to show that the RNA helicase Dbp7 is key player during early 60S ribosomal assembly. Dbp7 regulates a series of events driving compaction of domain V/VI in early pre60S ribosomal particles.

## Introduction

Ribosomes are essential macromolecular machines that decode messenger RNAs (mRNAs) and synthesize cellular proteins. The assembly of 79 ribosomal proteins (r-proteins) and four ribosomal RNAs (rRNAs) into functional small and large subunits (SSU/40S and LSU/60S, respectively) is a highly complex and energy-demanding cellular process^1–4^. Accurate and efficient assembly of these ribonucleoprotein complexes is therefore critical to ensure integrity of the proteome and allow cellular proliferation. Structural and biochemical analyses of ribosomes revealed that not only do the rRNAs fold to form intricate tertiary structures onto which the r-proteins assemble but also that rRNA moieties are essential for mRNA decoding and the catalytic action of ribosomes in peptide bond formation^1^. In the yeast *Saccharomyces cerevisiae*, nucleolar rDNA transcription leads to the production of a large 35S precursor rRNA (pre-rRNA) containing the sequences of the mature 18S, 5.8S, and 25S rRNAs. As the nascent pre-rRNA transcript is synthesized, a subset of r-proteins and numerous assembly factors (AFs) associate to form the SSU processome and 90S pre-ribosomal complexes^5^. Pre-rRNA processing to remove the internal and external transcribed spacers (ITS and ETS, respectively), chemical modification of rRNAs and rRNA folding then occur concomitant with the recruitment of further r-proteins and the dynamic association and dissociation of AFs^6–8^. During the ribosome assembly process, the pre-rRNA undergoes numerous structural transitions to achieve the final folding observed in mature ribosomes. Recent evidence from bacteria highlighted a role of transient interactions of r-proteins in regulating rRNA folding and in eukaryotes a subset of small nucleolar ribonucleoprotein complexes (snoRNPs) are proposed to act as rRNA chaperones^9–18^. In contrast to canonical snoRNPs that direct rRNA 2′-*O*-methylation, pseudouridylation, or acetylation^19^, these snoRNPs are typically non-catalytic and instead function by basepairing with specific rRNA sequences to tether them in particular conformations or to limit formation of aberrant rRNA-rRNA interactions.

Despite a wealth of recent structural insights into late pre-40S, late and intermediate pre-60S and SSU processome complexes, and functional analyses of the AFs present in these particles^20–34^, the early stages of 60S biogenesis remain something of a “black box”. In its final architecture, the 60S subunit has six defined structural domains (I to VI from 5′ to 3′) connected to each other by clustering of root helices found at the beginning of each domain^1^. It is believed that the earliest assembly steps involve the association of early r-proteins and AFs to the solvent-exposed structural domains I and II, leading to the folding and stabilization of these domains followed by those of domain VI^3,35,36^. This results in the formation and clustering of the respective root helices, which is an essential pre-requisite for subsequent maturation steps including downstream processing of the LSU pre-rRNAs and recruitment of later r-proteins. Several studies have described how large AFs, such as Rrp5 and Npa1, can act as rigid scaffolds and binding platforms for other AFs during early pre-60S biogenesis to help promote initial compaction and stabilization^37–40^. A key event during early pre-60S biogenesis is recruitment of uL3 (formerly Rpl3) as binding of this r-protein is crucial for the stabilization of earliest pre-60S intermediates and incorporation of all other 60S ribosomal proteins^35,36,41–45^. To date, mechanistic details of how AFs and early r-proteins mediate early compaction events as well as the interplay among them are lacking.

Approximately 30 AFs are known to transiently associate with early pre-60S particles, including eight RNA helicases (Dbp3, Dbp6, Dbp7, Dbp9, Has1, Mak5, Prp43, and Drs1)^46^. Through their functions in NTP-dependent remodeling of RNAs, RNA helicases often act as the driving force for structural transitions within RNP complexes. So far, Prp43 and Dbp3 are implicated in promoting the release of subsets of snoRNPs from pre-ribosomes^47,48^ whereas Mak5 and Has1 have recently been shown to be required for recruitment of specific r-proteins and AFs, respectively^49,50^. Interestingly, three of the relatively uncharacterized RNA helicases implicated in pre-60S biogenesis, Dbp6, Dbp7, and Dbp9, display genetic interactions among each other and with components of the Npa1 complex (composed of Npa1, Npa2, Nop8, and Rsa3) as well as uL3, suggesting that they may function cooperatively^44,45,51–53^. A recent study provided evidence that the Npa1 complex functions with Dbp6 to facilitate early clustering of root helices^38^, however, potential roles of other DEAD-box RNA helicases in early pre-60S compaction and their functional link with uL3 remained unexplored.

In this work, we reveal that Dbp7 binds to domain V/VI of 25S rRNA, close to the binding site of uL3 in early nucleolar pre-60S or 90S particles. Consistent with this, lack of Dbp7 results in inefficient recruitment of uL3 to pre-60S particles and pre-ribosomal accumulation of snoRNAs with target sites close to the Dbp7 binding site including the chaperone snoRNA snR190. Together, our data support pre-rRNA remodeling by Dbp7 to release snR190, a subset of other snoRNAs and the Npa1 complex from early pre-60S particles, thereby allowing incorporation of uL3 to stabilize domain I and VI interactions and license downstream 60S maturation events. Our findings provide important insights into how early remodeling events are coordinated by AFs to initiate compaction of the nascent 60S.

## Results

### Dbp7 is an RNA-dependent ATPase important for cell growth

**Fig. 1:**
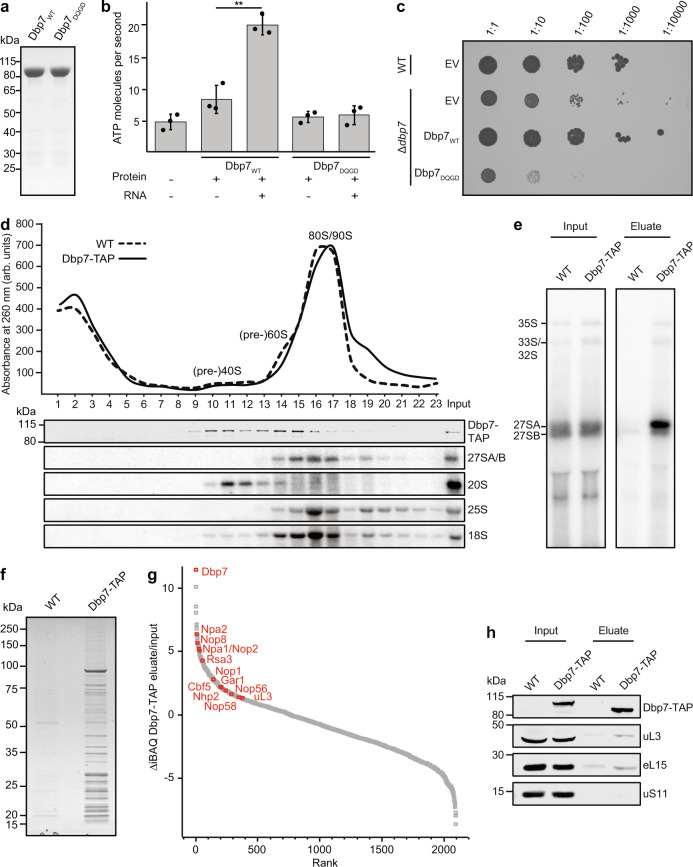
The RNA-dependent ATPase Dbp7 associates with early pre-60S complexes and is important for cell growth. **a** Equal amounts of His-ZZ-tagged Dbp7_WT_ and Dbp7_DQGD_ were separated by denaturing PAGE and proteins were visualized by Coomassie staining. Proteins from a single round of purification are shown. **b** The ATPase activity of purified His-ZZ-tagged Dbp7_WT_ and Dbp7_DQGD_ was monitored in vitro using 1.5 μM of each purified protein with (+) or without (−) 2.0 μM of RNA. Data are presented as mean ± standard deviation from *n* = 3 independent measurements. Significance was determined using unpaired, two-tailed Student’s *t*-test (Dbp7_WT_ + RNA vs −RNA *p* = 0.001792; **p < 0.01). **c** Equal numbers of cells from the Δ*dbp7* strain complemented with an empty pRS415 plasmid (EV) or pRS415-based plasmids for expression of Dbp7_WT_ or Dbp7_DQGD_ were serially diluted and spotted onto a selective plate. An equal amount of wild-type yeast (WT) carrying the EV was grown in parallel as a control. Growth was documented after 48 h of incubation at 30 °C. **d** Whole-cell lysates from wild-type yeast or the Dbp7-TAP strain were separated by using sucrose density centrifugation. Peaks corresponding to (pre-)ribosomal complexes are marked on an absorbance profile at 260 nm (top). Indicated (pre-)rRNAs and proteins in each fraction of the gradient were detected by northern and western blotting, respectively. **e** Extracts from wild-type yeast or a strain expressing TAP-tagged Dbp7 were used for pulldown assays on IgG sepharose. Co-purified pre-rRNAs were extracted and visualized by northern blotting using a mixture of probes hybridizing in ITS1 and ITS2. Input represents 3%. **f** Proteins present in the eluates of pulldowns performed as in (**e**) were separated by denaturing PAGE and visualized by Coomassie staining. **g** Proteins in input and eluate samples of pulldowns performed as in (**e**) were analyzed by mass spectrometry. Ranked differences in intensity-based absolute quantification (iBAQ) values between the input and eluate of the Dbp7-TAP sample are shown. Two independent experiments were performed and representative data is presented. **h** Pulldowns were performed as in (**e**) and the Dbp7-TAP bait as well as uL3, eL15, and uS11 were detected in input and eluate samples by western blotting using an anti-PAP antibody and antibodies against the endogenous r-proteins. Input represents 2.5%. Experiments presented in (**c**), (**d**), (**e**), and (**h**) were performed in duplicate or triplicate and representative data are shown.

Dbp7 is a putative RNA helicase localized in the nucleolus and implicated in LSU biogenesis (Supplementary Fig. 1a)^52^, but its catalytic activity and function(s) during subunit assembly have remained poorly characterized. A hallmark of RNA helicases is their ability to hydrolyze ATP in an RNA-dependent manner^54^. To demonstrate that Dbp7 possesses such catalytic activity, His_10_-ZZ-tagged wild-type Dbp7 (Dbp7_WT_) and Dbp7 carrying a glutamate (E) to glutamine (Q) substitution with the conserved DExD motif (Dbp7_DQGD_) were recombinantly expressed in *Escherichia coli* (*E. coli*) and purified (Fig. 1a). The purified proteins were then used for in vitro NADH-coupled ATPase assays in the presence or absence of a model RNA substrate. In the absence of RNA, Dbp7_WT_, but not Dbp7_DQGD_, hydrolyzed ATP above the background level observed in the absence of protein (Fig. 1b). Furthermore, a significant increase in the ATPase activity of Dbp7_WT_, but not Dbp7_DQGD_, was observed upon addition of RNA, demonstrating that Dbp7 is an RNA-dependent ATPase.

Having confirmed that E to Q substitution within the DEGD sequence motif renders Dbp7 catalytically inactive in vitro, a yeast complementation system was generated to assess the requirement for the catalytic activity of Dbp7 in vivo. This complementation system was based on a Δ*dbp7* strain, which notably has a growth rate half that of wild-type yeast (Supplementary Table 1). The Δ*dbp7* strain was transformed with a low-copy number plasmid containing the coding sequence (CDS) of wild-type or catalytically inactive Dbp7 as well as the sequences encoding the endogenous *DBP7* promoter and terminator, or an empty vector (EV) as a control. Growth of the resulting strains was analyzed by spot-test, demonstrating that the growth defect caused by the absence of endogenous Dbp7 can be rescued by expression of plasmid-derived Dbp7_WT_ (Fig. 1c). Expression of Dbp7_DQGD_ resulted in a more pronounced growth retardation than lack of Dbp7 (Δ*dbp7* + EV) (Fig. 1c), implying that the presence of the catalytically inactive protein is more disadvantageous for cells than its absence. These results highlight an important cellular function of Dbp7 dependent on its catalytic activity.

### Dbp7 associates with early pre-ribosomal particles

To explore the role of Dbp7 in ribosome assembly, the association of Dbp7 with pre-ribosomal particles was first analyzed in sucrose density gradients. Whole-cell extracts from wild-type yeast or a strain expressing Dbp7-TAP, which grows as the wild-type strain (Supplementary Fig. 1b; Supplementary Table 1), were separated by sucrose density gradient centrifugation. Profiles of absorbance at 260 nm indicated the migration positions of (pre-)40S, (pre-)60S, and 80S ribosomal particles, which were confirmed by the detection of specific (pre-)rRNAs present in each of these complexes (Fig. 1d). Western blotting revealed that Dbp7-TAP was enriched in fractions containing (pre-)60S complexes supporting a role of Dbp7 in the 60S biogenesis but was also detectable co-migrating with (pre-)40S subunits (Fig. 1d).

To gain further insight into the pre-ribosomal particles Dbp7 associates with, different pre-60S particles were isolated from yeast strains expressing Dbp7-GFP and affinity-tagged AFs. Dbp7-GFP was detected in pre-60S particles isolated via early (Npa1, Npa2, Ssf1, Nip7, Nop2) and intermediate (Erb1 and Rpf2) factors, but was not detected in late pre-60S containing Arx1 (Supplementary Fig. 1c). The RNA and protein compositions of pre-ribosomal particles co-purified with Dbp7-TAP from whole-cell lysates were also determined. Extracts from wild-type yeast were analyzed in parallel to control for specific enrichment and detection of pre-rRNA intermediates in input samples from wild-type and Dbp7-TAP cells verified no difference in pre-rRNA processing upon C-terminal tagging of Dbp7 (Fig. 1e). Co-purified pre-rRNAs were detected by northern blotting using a mixture of probes hybridizing in the ITS1 and ITS2 pre-rRNA regions. This revealed strong enrichment of the 27SA pre-rRNA species with Dbp7-TAP as well as weaker enrichment of the 35S and 33S/32S intermediates (Fig. 1e), confirming the physical association of Dbp7 with early pre-60S particles.

The compositions of the pulldown inputs and eluates were also analyzed using Coomassie blue staining and mass spectrometry (MS) (Fig. 1f, g). Consistent with the presence of Dbp7-GFP in early pre-60S particles (Supplementary Fig. 1c), plotting the difference in intensity-based absolute quantification (iBAQ) values between inputs and eluates of both the Dbp7-TAP and wild-type pulldown samples, indicated the enrichment of r-proteins and AFs that associate with very early, nucleolar pre-60S particles, including components of the Npa1 complex and uL3 (Fig. 1g, h; Supplementary Fig. 1d; Source data) with Dbp7-TAP. These results demonstrate that the previously identified genetic interaction network between Dbp7 and these proteins^53^ likely reflects a physical association and provide compelling evidence for a functional role of Dbp7 with these proteins during early pre-60S biogenesis. Interestingly, components of the box C/D and H/ACA snoRNPs were also found to be enriched with Dbp7-TAP, suggesting that Dbp7 is recruited to pre-60S particles before the process of snoRNP-guided rRNA modification is completed and/or while snoRNPs chaperoning pre-rRNA folding are present. Collectively, these results demonstrate that Dbp7 associates with very early pre-60S particles and suggest potential roles for Dbp7 in regulating snoRNP dynamics on pre-ribosomes and/or in mediating the initial compaction of the nascent 60S together with the Npa1 complex.

### Specific sites of rRNA 2′-*O*-methylation are mildly affected by lack of Dbp7

**Fig. 2:**
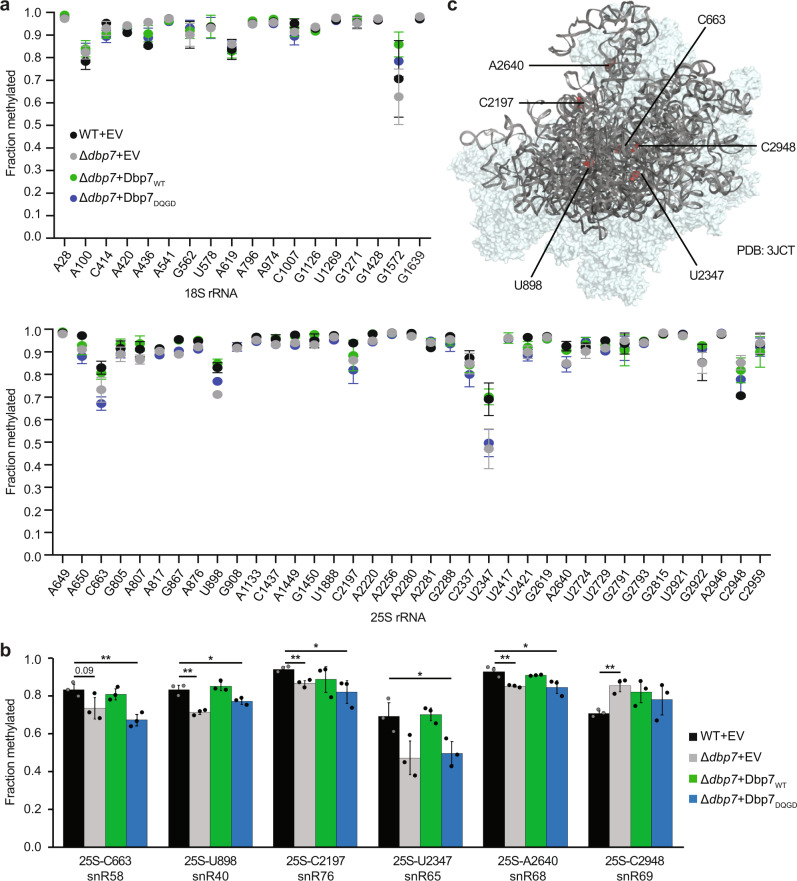
Dbp7 is required for efficient 2′-*O*-methylation of specific rRNA nucleotides. **a** Total RNA extracted from exponentially growing cells from the strains of the Δ*dbp7* complementation system was used for RMS analysis. Experiments were performed in triplicate (see Source data for individual data points) and the mean RMS score plotted for each 2′-*O*-methylated nucleotide in the 25S and 18S rRNAs. Error bars represent standard deviation. **b** Bar graphs of the RMS scores for each of the four strains at sites where the extent of 2′-*O*-methylation varies significantly are shown. Data from *n* = 3 independent measurements are presented as mean ± standard deviation. Statistical significance was calculated using unpaired, two-tailed Student’s *t*-test (C663 WT + EV vs Δ*dbp7* + EV *p* = 0.09432, WT + EV vs Δ*dbp7* + Dbp7_DQGD_ *p* = 0.005907; U898 WT + EV vs Δ*dbp7* + EV *p* = 0.00289, WT + EV vs Δ*dbp7* + Dbp7_DQGD_ *p* = 0.037832; C2197 WT + EV vs Δ*dbp7* + EV *p* = 0.005727, WT + EV vs Δ*dbp7* + Dbp7_DQGD_ *p* = 0.049087; U2347 WT + EV vs Δ*dbp7* + Dbp7_DQGD_ *p* = 0.043687; A2640 WT + EV vs Δ*dbp7* + EV *p* = 0.007783, WT + EV vs Δ*dbp7* + Dbp7_DQGD_ *p* = 0.048903; C2948 WT + EV vs Δ*dbp7* + EV *p* = 0.004162; *p < 0.05, **p < 0.01). **c** The positions of nucleotides carrying 2′-*O*-methylations significantly affected by lack of Dbp7 are indicated on a pre-60S particle purified via TAP-tagged Nog2 (PBD:3JCT)^25^.

The finding that Dbp7 is associated with early pre-60S particles containing snoRNPs raised the possibility that Dbp7 may contribute to efficient snoRNA-guided pre-rRNA modification. To explore whether Dbp7 and its catalytic activity regulate rRNA 2′-*O*-methylation, RiboMeth-seq (RMS)^55,56^ was employed to monitor the extent of 2′-*O*-methylation of each rRNA nucleotide in the established Dbp7 complementation system. Lack of Dbp7 resulted in mild, but significant, changes in the extent of 2′-*O*-methylation of some nucleotides within the 25S rRNA (C663, U898, C2197, U2347, A2640, and C2948), while methylation of the 18S rRNA was unaffected (Fig. 2a, b and Source data). In all cases, except C2948, the RMS score was lower in the absence of Dbp7 than in wild-type yeast. These mild methylation defects were rescued by expression of Dbp7_WT_ from a plasmid, whereas expression of Dbp7_DQGD_ caused similar alteration of rRNA 2′-*O*-methylation to lack of Dbp7. Mapping the affected rRNA 2′-*O*-methylation sites onto the tertiary structure of the 25S rRNA in a pre-60S particle purified via Nog2 (PBD:3JCT)^25^ did not reveal spatial clustering of these modifications consistent with direct regulation of methylation by Dbp7 bound to a specific pre-60S region (Fig. 2c). This rather suggests that the effects on 25S rRNA 2′-*O*-methylation likely arise as an indirect consequence of Dbp7 action.

### Pre-rRNA processing and rRNA production are impaired in cells lacking Dbp7 activity

**Fig. 3:**
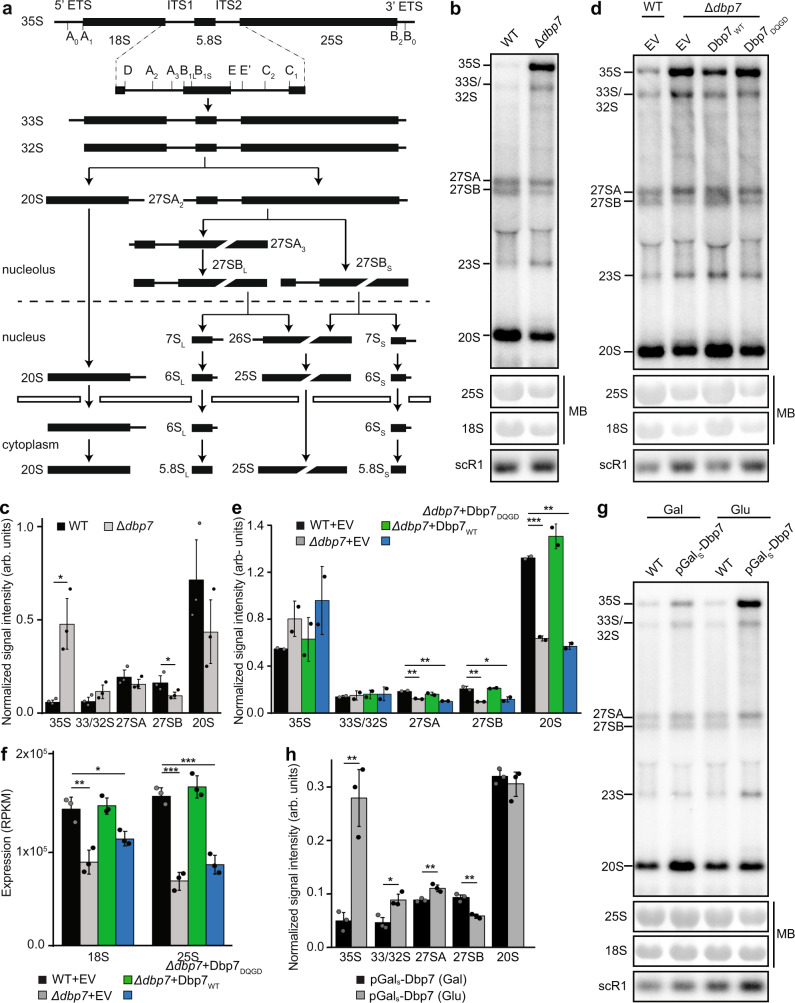
Pre-rRNA processing is impaired in cells lacking the remodeling activity of Dbp7 and pre-rRNAs are degraded. **a** Schematic outline of pre-rRNA processing in *S. cerevisiae*. Mature rRNA sequences are represented as black rectangles, and the internal transcribed spacers (ITS) and external transcribed spacers (ETS) are shown by black lines. Pre-rRNA cleavage sites are indicated on the 35S pre-rRNA transcript. **b** Total RNA extracted from wild-type yeast (WT) and the Δ*dbp7* strain was analyzed by northern blotting using probes hybridizing in ITS1 and ITS2 to detect the indicated pre-rRNA species. Mature 25S and 18S rRNA were visualized by methylene blue (MB) staining. **c** The levels of pre-rRNA intermediates in *n* = 3 independent experiments performed as in (**b**) were quantified and normalized according to the amount of the scR1 RNA. Data are represented as mean ± standard deviation and significance was determined using unpaired, two-tailed Student’s *t*-test (35S WT vs Δ*dbp7* *p* = 0.012174; 27SB WT vs Δ*dbp7* *p* = 0.046764; *p < 0.05). **d** Pre-rRNA processing was analyzed in the Δ*dbp7* strain carrying an empty pRS415 plasmid (EV) or pRS415-based plasmids for expression of Dbp7_WT_ or Dbp7_DQGD_ and in wild-type yeast (WT) carrying the EV as in (**b**). **e** The levels of pre-rRNA intermediates in *n* = 3 independent experiments performed as in (**d**) were quantified as in (**c**) and significance was determined using unpaired, two-tailed Student’s *t*-test (27SA WT + EV vs Δ*dbp7* + EV *p* = 0.003716, WT + EV vs Δ*dbp7* + Dbp7_DQGD_ *p* = 0.002347; 27SB WT + EV vs Δ*dbp7* + EV *p* = 0.016287, WT + EV vs Δ*dbp7* + Dbp7_DQGD_ *p* = 0.025775; 20S WT + EV vs Δ*dbp7* + EV *p*=0.000899, WT + EV vs Δ*dbp7* + Dbp7_DQGD_ *p* = 0.00111; *p < 0.05, **p < 0.01, ***p < 0.001). **f** The normalized (reads per kilobase of transcript, per million mapped reads; RPKM) numbers of sequencing reads mapping to the 18S and 25S rRNAs was determined in the RMS datasets for wild-type yeast containing an empty vector (EV) and the *Δdbp7* strain complemented with EV or plasmids for expression of Dbp7_WT_ or Dbp7_DQGD_. Relative rRNA levels from *n* = 3 biologically independent RMS experiments in shown as mean ± standard error and significance was determined using unpaired, two-tailed Student’s *t*-test (18S WT + EV vs Δ*dbp7* + EV *p* = 0.005626, WT + EV vs Δ*dbp7* + Dbp7_DQGD_ *p* = 0.020001; 25S WT + EV vs Δ*dbp7* + EV *p* = 0.000275, WT + EV vs Δ*dbp7* + Dbp7_DQGD_ *p* = 0.000685; *p < 0.05, **p < 0.01, ***p < 0.001). **g** Pre-rRNA processing in wild-type yeast (WT) and the pGAL_S_-3HA-Dbp7 strain grown in medium containing galactose (Gal) or glucose (Glu) for 12 h was analyzed as in (**b**). **h** The levels of pre-rRNA processing intermediates in *n* = 3 independent experiments performed as in (**g**) were quantified as in (**c**). Data are presented as mean ± standard deviation and significance was determined using unpaired two-tailed Student’s *t*-test (35S *p* = 0.004256, 33/32S *p* = 0.014288, 27SA *p* = 0.008295, 27SB *p* = 0.001472; *p < 0.05, **p < 0.01).

The mild effects on rRNA 2′-*O*-methylation observed by RMS are unlikely to cause the strong growth defects observed in cells lacking Dbp7 or its catalytic activity. Therefore, pre-rRNA processing and the levels of the mature rRNAs (Fig. 3a) were examined in the Δ*dbp7* strain and the complementation system. As previously observed^52^, northern blotting analyses demonstrated that compared to wild-type yeast, the Δ*dbp7* strain showed accumulation of the 35S pre-rRNA, and decreases in the levels of the 27SA, 27SB, and 20S species (Fig. 3b, c). Similar pre-rRNA processing defects were also observed in the Δ*dbp7* strain carrying an empty vector compared to its equivalent control, and were rescued by expression of plasmid-derived Dbp7_WT_ but not Dbp7_DQGD_ (Fig. 3d, e) demonstrating the requirement of the catalytic activity of Dbp7 for pre-rRNA processing.

Strikingly, although similar amounts of total RNA were analyzed, reduced levels of the mature 25S and 18S rRNAs were observed in cells lacking Dbp7 or its catalytic activity (Fig. 3b, d). To quantify the effects on rRNA production, the relative numbers of sequencing reads mapping to rRNAs were determined in the available RMS datasets. This revealed a significant decrease in the amount of 18S rRNA present and an even more pronounced reduction in the 25S rRNA level in cells lacking Dbp7 that was restored by expression of Dbp7_WT_ but not Dbp7_DQGD_ (Fig. 3f). The finding that the amount of mature rRNAs, which represent ~80% of cellular total RNA, are reduced in the absence of Dbp7 or its catalytic activity is further supported by the observation that the relative expression of other classes of RNAs, for example, tRNAs, are increased in cells lacking Dbp7 or expressing Dbp7_DQGD_ compared to wild-type yeast or the Δ*dbp7* strain complemented with Dbp7_WT_ (Supplementary Fig. 2a).

To better discriminate between changes in pre-rRNA levels arising from defective pre-rRNA processing and those occurring due to turnover of pre-rRNAs, a system for transient depletion of Dbp7 was generated in which expression of *DBP7* is under the control of a truncated galactose-inducible and glucose-repressible promoter (pGAL_S_). The expression level of 3HA-Dbp7 from this promoter is elevated compared to the level of Dbp7-3HA expressed from the endogenous promoter (Supplementary Fig. 2b), however, pre-rRNA processing in the pGAL_S_-3HA-Dbp7 strain was comparable to that in wild-type yeast when grown in galactose-containing media (Fig. 3g). Expression of 3HA-Dbp7 was reduced after 6 h growth in glucose-containing media and was undetectable after 10 h (Supplementary Fig. 2c). Growth of the pGAL_S_-3HA-Dbp7 strain was comparable in glucose-containing and galactose-containing media for 12 h (Supplementary Table 1) and importantly, mature rRNA levels were unaltered (Fig. 3g). In this context, depletion of Dbp7 lead to significant accumulation of the 35S pre-rRNA and the 33S/32S and 27SA processing intermediates as well as reduced 27SB, but the 20S species was unaffected (Fig. 3g, h).

Taken together, these results demonstrate that the remodeling activity of Dbp7 is required for the efficient conversion of the 27SA pre-rRNAs into 27SB via processing at the A_2_, A_3_, and B_1L/S_ sites. The reduced levels of the 27SA and 20S processing intermediates observed in the Δ*dbp7* strain likely arise indirectly due to turnover of upstream pre-rRNAs. Furthermore, in the absence of the remodeling activity of Dbp7, early pre-rRNA transcripts may be degraded leading to reduced production of mature rRNAs and likely, the observed growth defects of the Δ*dbp7* strain. This is in line with pulse-chase labeling of nascent (pre-)rRNAs, which revealed delayed 25S rRNA production but no accumulation of early pre-rRNA in cells lacking Dbp7^52^.

### Dbp7 cross-links to specific sites within domains V and VI of the 25S rRNA

**Fig. 4:**
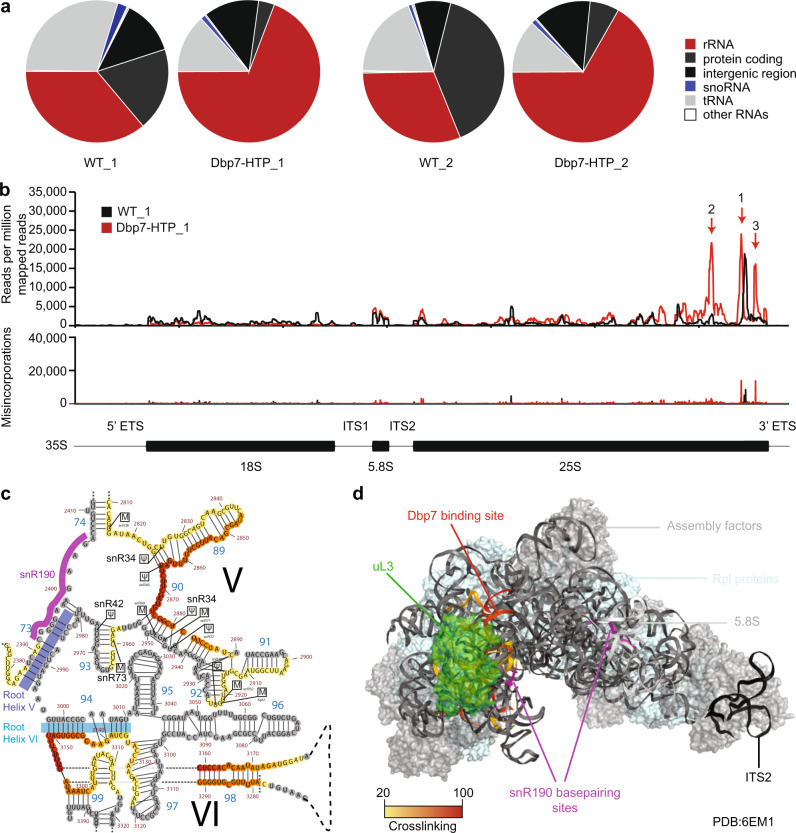
Dbp7 cross-links to domain V and VI of the 25S rRNA sequence. **a** The relative numbers of reads mapping to genes encoding different RNA species in the wild-type control (WT) and Dbp7-HTP PAR-CRAC samples are presented as pie charts. Highlighted in red and blue are the proportions of reads mapping to the rDNA locus and snoRNA genes, respectively. Data from two independent PAR-CRAC experiments are shown. **b** The normalized numbers of reads mapping to each nucleotide of the *RDN37* rDNA transcription unit in the Dbp7-HTP and WT samples are shown in the upper panel. The normalized number of the T-to-C misincorporations mapping to each nucleotide of *RDN37* is shown in the lower panel. A schematic representation of the 35S pre-rRNA transcript is shown at the bottom with black rectangles corresponding to the mature rRNAs and black lines representing the transcribed spacers. **c** A magnified view of the domain V and VI regions of the 25S rRNA secondary structure, containing the three major Dbp7-HTP cross-linking sites is shown. The number of sequencing reads mapping to each nucleotide of 25S rRNA is represented by a color code where the maximum number of reads (100%) is shown in red and lower numbers of reads (down to 20%) are shown in orange-yellow. The predicted basepairing site of snR190 is indicated by a purple line, and the domain V and VI root helices are indicated in blue and cyan, respectively. **d** Modeling of the identified Dbp7-HTP cross-linking sites onto a tertiary structure of the 25S rRNA in the state C nucleolar pre-60S particle purified via TAP-tagged Nsa1 followed by Flag-tagged Ytm1 (PDB: 6EM1) using a color scale as in (**c**). The density corresponding to uL3 is colored in green and snR190 basepairing sites are indicated in purple. In panels (**b**–**d**), data from a single, representative PAR-CRAC dataset is shown.

The pre-rRNA processing defects and degradation of pre-rRNAs in the absence of Dbp7 catalytic activity, together with the strong growth defect observed in the Δ*dbp7*, indicate that remodeling of early pre-60S particles by Dbp7 represents an important step during ribosome assembly. Key to elucidating the functions of RNA helicases within large RNPs is identifying their binding site(s). The in vivo photoactivatable ribonucleoside-enhanced cross-linking and analysis of cDNA (PAR-CRAC) approach^57–59^ was therefore employed to discover RNA sequences bound by Dbp7 in cells. Wild-type yeast and a strain expressing C-terminally His_6_-TEV protease cleavage site-Protein A (HTP)-tagged Dbp7 were grown in the presence of 4-thiouracil (4sU) before cross-linking using light at 365 nm. Protein–RNA complexes were purified under native and denaturing conditions and co-purified RNAs were partially digested to leave intact only fragments directly bound by proteins. Co-purified RNA fragments were copied into a cDNA library that was subjected to Illumina deep sequencing. The obtained sequencing data were mapped onto the *Saccharomyces cerevisiae* genome using a filter to retain only reads containing specific T-to-C misincorporations that are introduced during reverse transcription of RNAs containing 4sU. Two independent PAR-CRAC experiments were performed and, in both cases, analysis of the distribution of sequencing reads derived from different classes of RNAs revealed a larger portion of reads (67/69%) originating from rRNAs in the Dbp7-HTP PAR-CRAC dataset compared to the wild-type dataset (31/36%) (Fig. 4a). The fragments present in the wild-type control sample reflect non-specific, background RNAs that are co-purified during the PAR-CRAC procedure and the substantial enrichment of reads mapping to the rDNA in the Dbp7-HTP dataset supports the involvement of Dbp7 in ribosome biogenesis.

Due to the high reproducibility of the PAR-CRAC results, a single, representative dataset is presented for the subsequent analyses. Analysis of the normalized numbers of sequencing reads mapping to each nucleotide of the *RDN37* locus encoding the initial 35S pre-rRNA transcript revealed three prominent peaks (peaks 1–3) close to the 3′ end of the 25S rRNA, representing Dbp7-HTP cross-linking sites (Fig. 4b, upper panel). RNA fragments corresponding to these three cross-linking sites were not enriched in the control sample and the cross-linking sites are also specific when compared to the binding sites of other RNA helicases previously analyzed^18,47,49^. Binding of Dbp7 to this region is further supported by the presence of nucleotide misincorporations (Fig. 4b, lower panel). The two-dimensional arrangement of these distinct peaks was then examined by modelling them on the secondary structure of mature 25S rRNA^60^. This revealed that the Dbp7-HTP cross-linking sites lie in helix 90 of domain V of the 25S rRNA and helices 94, 98, and 99 of domain VI (Fig. 4c). These cross-linking sites correlate well with a function of Dbp7 in the early events of 60S biogenesis as domain VI, together with domains I and II, are the first regions to display rRNA folding and stabilization^3,25,29,35^ and helix 94 is a domain VI root helix. Interestingly, both strands of helices 98 and 99, which are separate by a 100-nucleotide loop sequence, are cross-linked by Dbp7-HTP. This suggests that the basepairing of helices 98 and 99 observed in mature ribosomes is already established during the initial steps of pre-60S biogenesis (Fig. 4c). To date, Dbp7 is not present in any available cryo-EM structure of a pre-60 particle^21,23,25,27–32^. As our data suggest that Dbp7 acts during the early 60S assembly steps, the Dbp7-HTP cross-linking sites identified by PAR-CRAC were mapped onto the tertiary structure of the 25S rRNA in the earliest available pre-60S particles^25^. Among the different nucleolar pre-60S structures available, the three Dbp7-HTP prominent cross-linking sites can only be completely visualized on the state C particle (PDB:6EM1) purified via TAP-tagged Nsa1 followed by Flag-tagged Ytm1. The Dbp7-HTP cross-linking sites cluster in close proximity on this particle, supporting the notion that they reflect different contact points of a single Dbp7 binding event (Fig. 4d). Visualization of the r-proteins present in the vicinity of the Dbp7-HTP cross-linking site revealed the close proximity of uL3, consistent with the strong enrichment of this r-protein in Dbp7-containing pre-60S particles (Figs. 1g, h and 4d).

### A subset of snoRNAs accumulates on pre-60S particles when Dbp7 is lacking

**Fig. 5:**
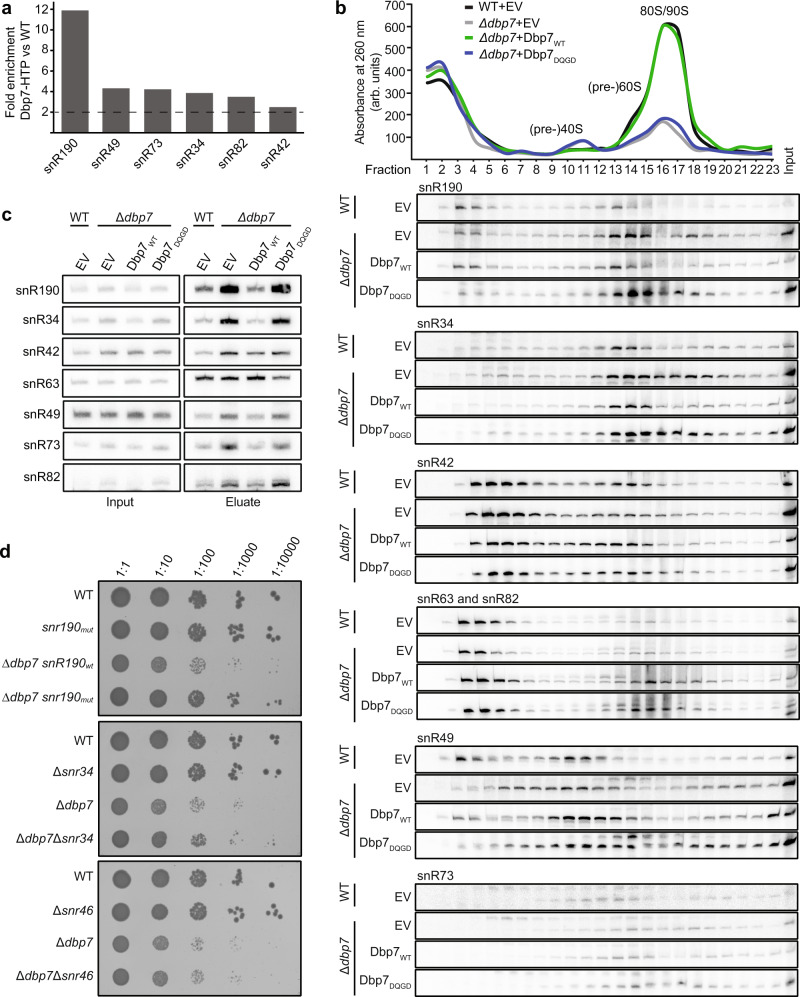
A subset of snoRNAs accumulate on pre-60S particles in the absence of Dbp7. **a** The fold enrichments of snoRNAs showing >two-fold enrichment in Dbp7-HTP_2 PAR-CRAC dataset compared to WT_2 are shown. **b** Whole-cell extracts from wild-type yeast (WT) containing an empty vector (EV) and the Δ*dbp7* strain carrying EV or plasmids for expression of Dbp7_WT_ or Dbp7_DQGD_ were separated by sucrose density gradient centrifugation. RNAs extracted from each fraction were separated by denaturing PAGE and the distributions of the indicated snoRNAs were analyzed by northern blotting. Where snR63 and snR82 are simultaneously detected, the lower band corresponds to snR63 and the upper to snR83. Experiments were performed in duplicate and representative data is shown. **c** Extracts from yeast strains expressing Nop2-TAP in the background of the Dbp7 complementation system were used for pulldowns on IgG sepharose. RNAs present in inputs (3%) and eluates were extracted, separated by denaturing PAGE and the indicated snoRNAs detected by northern blotting. Experiments were performed in duplicate and representative data is shown. **d** Equal numbers cells from the indicated yeast strains were serially diluted and spotted onto a plate. Growth was documented after 72 h of incubation at 30 °C.

Analysis of the distribution of PAR-CRAC sequencing reads between different classes of RNA also revealed enrichment of reads mapping to snoRNA sequences in the Dbp7-HTP datasets compared to the control (Fig. 4a). As the identified Dbp7 cross-linking site on the 25S rRNA is close to the pre-rRNA basepairing sites of several snoRNAs (Fig. 4c) and snoRNP proteins were co-purified with Dbp7-TAP (Fig. 1g), the identities of the snoRNAs enriched in the Dbp7-HTP PAR-CRAC data were investigated. This revealed a more than two-fold enrichment of several snoRNAs (snR190 (C/D), snR49 (H/ACA), snR73 (C/D), snR34 (H/ACA), snR82 (H/ACA), and snR42 (H/ACA)) relative to the wild-type control (Fig. 5a). Strikingly, snR190, which is 12-fold enriched in the Dbp7-HTP PAR-CRAC data, is suggested to chaperone rRNA folding rather than guiding rRNA 2′-*O*-methylation^61^ and has a pre-rRNA basepairing site in close proximity to the identified Dbp7-HTP pre-rRNA cross-linking sites. Notably, several, but not all of the other enriched snoRNAs, also have basepairing sites in the vicinity of the Dbp7-HTP pre-rRNA cross-linking sites. The snoRNAs guiding the rRNA 2′-*O*-methylations affected by lack of Dbp7 (Fig. 2a, b) were not found to be enriched, further supporting the conclusion that the defects in rRNA 2′-*O*-methylation observed in cells lacking Dbp7 or its catalytic activity are long-range, indirect effects of impaired pre-60S remodeling. To confirm the presence of the enriched snoRNAs in Dbp7-containing pre-ribosomes, a pulldown was performed using Dbp7-TAP and snoRNAs were detected by northern blotting. snR190, snR73, snR82, and snR42 were strongly enriched in Dbp7-containing particles, and snR34 and snR49 were also present above background levels (Supplementary Fig. 3a). The lesser association of snR34 and snR49 with Dbp7-TAP could reflect a weak or transient association of these snoRNAs with pre-60S particles, meaning that they are better captured with cross-linking. The specific enrichment of only a subset of snoRNAs in Dbp7-containing pre-60S particles is further supported by the finding that snR63, which was not enriched in the Dbp7-HTP PAR-CRAC data, was also not enriched above background in the Dbp7-TAP pulldown.

To explore whether lack of Dbp7 affects the dynamics of these snoRNAs on pre-ribosomes, whole-cell lysates from wild-type yeast and the Δ*dbp7* strain carrying either an empty vector or a plasmid for expression of Dbp7_WT_ or Dbp7_DQGD_ were subjected to sucrose density centrifugation. Monitoring the absorption profiles at 260 nm confirmed a strong reduction in 60S subunit production, relative to 40S, and a concomitant decrease in 80S monosomes in cells lacking Dbp7 or its catalytic activity (Fig. 5b; upper panel). The distribution of selected snoRNAs was then analyzed by northern blotting (Fig. 5b). The levels of snR42 on pre-ribosomal complexes were not strongly affected by lack of Dbp7 or its activity. However, in cells lacking Dbp7 or its activity, the amount of snR190 present on large pre-ribosomal complexes (fractions 17–23) was higher than in cells expressing wild-type Dbp7, while the amount of snR190 present in non-ribosomal complexes (fractions 1–7) was reduced, suggesting accumulation of snR190 in early/pre-60S complexes in the absence of the remodeling activity of Dbp7. Likewise, for snR34, a notable increase in the amount of the snoRNA on pre-60S and larger pre-ribosomal complexes (fractions 13–23) was observed in cells lacking Dbp7 or its catalytic activity compared to wild-type.

As these data indicate a role for Dbp7 in modulating snoRNA levels on pre-60S complexes, the levels of a subset of snoRNAs were monitored in pre-60S particles isolated from wild-type yeast and the strain lacking Dbp7. The AF Nop2 is present in the state D and E cryo-EM structures of nucleolar 60S precursors, where it is positioned far away from the Dbp7-binding sites in domain V/VI^25^. Nop2 was also identified in Dbp7-containing particles (Fig. 1g) and this association was confirmed by a reverse pulldown via Nop2-TAP followed by western blotting analysis (Supplementary Fig. 3b). Analysis of the pre-rRNAs associated with Nop2 revealed a strong enrichment of the 27SB pre-rRNA (Supplementary Fig. 3c), implying that although Dbp7 and Nop2 are both present in common pre-60S complexes, Nop2 remains associated after dissociation of Dbp7. Collectively, these observations highlight pre-60S particles purified via Nop2 as ideal to investigate changes in the 60S maturation pathway when Dbp7 is lacking. Pre-60S particles were therefore isolated via Nop2-TAP from cells expressing Dbp7 and those lacking Dbp7 or its catalytic activity, and northern blotting was used to detect selected snoRNAs. Consistent with the gradient analysis, this revealed accumulation of snR190 and snR34 in Nop2-containing particles in cells lacking Dbp7 or its catalytic activity compared those expressing wild-type Dbp7, while the levels of other snoRNAs (e.g., snR42) were not so strongly affected (Fig. 5c).

snR34 guides pseudouridylation of 25S-U2826 and 25S-U2880 in close proximity to the Dbp7 cross-linking sites so we investigated whether the altered dynamics of this snoRNA on pre-ribosomes in cells lacking Dbp7 or its catalytic activity affects modification of these sites. Primer extension was performed on CMC-labeled RNAs isolated from strains of the complementation system and strains lacking specific snoRNAs as control, however, no differences in pseudouridylation of the sites guided by snR34 or 25S-U2865 guided by snR46, a snoRNA not enriched with Dbp7, were observed (Supplementary Fig. 4a,b). snR190 was initially predicted to 2′-*O*-methylate 25S-G2395 close to the Dbp7 pre-rRNA cross-linking sites, but no methylation of this site has been detected^55,61,62^. Instead, predicted snR190 basepairing sites in domains I and V of the 25S rRNA have led to the suggestion that the snoRNA may function as a pre-rRNA folding chaperone (Fig. 4c, d)^38,61^. The distribution of sequencing reads mapping to snR190 (and snR34 and snR42) were examined to determine whether Dbp7 physically associates with the regions of snR190 involved in basepairing with the 25S rRNA. While Dbp7-HTP predominantly cross-linked to the 5′ end of snR34, in the case of snR190, the majority of reads mapped across the box B sequence that basepairs with domain I of the pre-rRNA, and close to the 3′ end of the snoRNA (Supplementary Fig. 5a-c). Interestingly, the cross-linking site of Dbp7-HTP on snR190 is distinct from a previously reported cross-linking site of Npa1^38^.

The finding that lack of Dbp7 compromises the release of snoRNAs with pre-rRNA target sites close to Dbp7-binding region, most notably snR190 and snR34, from pre-ribosomes raised the possibility that failure to release these snoRNAs underlies the impaired growth of the Δ*dbp7* strain (Fig. 1c; Supplementary Table 1). Yeast strains lacking *snR34* or *snR42*, or carrying a mutation in box C of snR190 that destabilizes the snoRNA, were therefore generated in the wild-type or Δ*dbp7* background (Supplementary Fig. 6a-d)^61^. Growth analysis of these strains demonstrated that lack of snR34, snR42, or snR190 did not strongly affect growth (Fig. 5d; Supplementary Table 1). Strikingly, lack of either snR190 or snR34, but not snR42, partially rescued the growth defect of the Δ*dbp7* strain (Fig. 5d), suggesting that Dbp7-induced release of these snoRNAs from pre-60S particles is an important step in LSU biogenesis.

### Dbp7 is required for efficient recruitment of “A3 factors” and uL3 to pre-60S particles

The degradation of pre-rRNAs in the Δ*dbp7* strain and the retention of a subset of snoRNAs, including snR190, on pre-60S complexes when Dbp7 is lacking, demonstrate that the initial stages of pre-60S biogenesis are fundamentally impaired in the absence of Dbp7. Release of snoRNAs in early pre-60S particles, especially snR190 which plays a role in compaction of domains I and V^38^, is likely a key early maturation event important for licensing downstream steps such as recruitment of r-proteins and/or AFs.

**Fig. 6:**
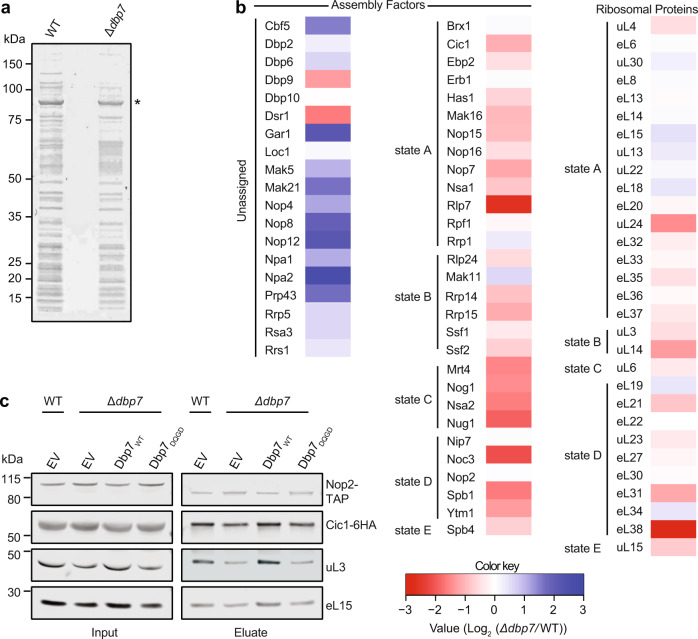
Dbp7 is required for the recruitment of uL3 to pre-ribosomes. **a**, **b** Nop2-TAP particles were isolated from whole-cell lysate of wild-type (WT) and Δ*dbp7* cells on IgG sepharose and eluted by overnight TEV cleavage. Associated proteins were separated by denaturing PAGE (**a**; asterisk indicates the Nop2 bait protein) and subjected to MS analysis (**b**). A heat map was generated from two independent biological replicates and shows the relative enrichment (log2 fold-change Δ*dbp7* vs. WT) of assembly factors and ribosomal proteins clustered based on their available cryo-EM data (PDB: 6EM3, 6EM4, 6EM1, 6EM5, 6ELZ, 6C0F, 6CB1)^25,29^. **c** Pre-60S particles were isolated via Nop2-TAP from yeast strains expressing Cic1-HA from its endogenous genomic locus in the context of the Dbp7 complementation system as in (**a**). The indicated proteins were detected in input and eluate samples by western blotting using antibodies against endogenous uL3 and eL15, the HA tag for Cic1, and the calmodulin-binding protein (CBP) for Nop2.

To assess the influence of Dbp7 on the protein composition of pre-60S particles, Nop2-containing particles were isolated from whole-cell lysate of wild-type and Δ*dbp7* cells, and their composition was analyzed using MS (Fig. 6a, b). Analysis of the obtained MS data revealed decreases in the amounts of many proteins present in the Nop2-containing pre-60S particle in the absence of Dbp7 (Fig. 6b; Supplementary Table 2). Among the AFs, strong decreases in the levels of several “A3 factors” (e.g., Rlp7, Cic1, Nop15, Nop7), which are key players in the processing of 27SA to 27SB, were observed^63^. This finding is in line with the pre-rRNA processing defects observed in the absence of Dbp7 (Fig. 3) and was consolidated by western blot analysis of the “A3 factor” Cic1, which was present at lower levels in Nop2-TAP particles enriched from cells lacking Dbp7 or its catalytic activity than on those isolated from cells expressing Dbp7 (Fig. 6b, c). Furthermore, DMS structure probing analysis of the ITS2 regions bound by these factors revealed differences in nucleotide accessibility in the presence and absence of Dbp7 consistent with the impaired recruitment of these proteins (Supplementary Fig. 7). Consistent with the hierarchical association of AFs in early 60S particles, decreases in the levels of several B-factors in Nop2-TAP particles purified from cells lacking Dbp7 were also observed (Fig. 6b, Supplementary Table 2).

In contrast, the levels of various AFs that participate in the earliest stages of 60S biogenesis (e.g., Npa1, Npa2, Nop8, Rsa3, Dbp6, Dbp9, Dbp3, Mak5, Rrp5, Nop4, Nop12, Prp43, Cbf5, Gar1), were found to be higher in Nop2-TAP particles isolated from the Δ*dbp7* strain than those purified from wild-type yeast. The retention of these AFs, many of which are present in Dbp7-TAP particles (Supplementary Table 2), in predominantly later Nop2-containing particles, is consistent with impeded maturation and/or incorrect assembly of very early pre-60S complexes when Dbp7 is lacking. Enrichment of the Npa1 complex (Npa1, Npa2, Nop8, Rsa3, and Dbp6) raises the possibility that Dbp7 contributes, directly or indirectly, to dissociation of this chaperone complex and the enrichment of the H/ACA snoRNP proteins Cbf5 and Gar1 is consistent with the observed accumulations of several H/ACA snoRNAs in Nop2-containing particles when Dbp7 is lacking (Fig. 5b, c). Altogether, these data indicate that the pre-ribosomes purified from Δ*dbp7* cells accumulate as very early assembly intermediates, highlighting a key role of Dbp7 in driving efficient maturation of early pre-60S.

Examination of ribosomal proteins present in Nop2-TAP particles purified from wild-type and Δ*dbp7* cells revealed generally milder effects than for the AFs (Fig. 6b, Supplementary Table 2). eL38, uL24, uL14, eL31, and eL21 were the most affected, with more than two-fold decreases observed in the absence of Dbp7 (Fig. 6b). These r-proteins are mostly only present in later nucleolar particles^25^ and their depletion causes accumulation of either the 27SB or 7S pre-rRNAs^35,41,43,64^. Notably, these r-proteins are all non-essential^41^. The lack of strong effects on r-proteins and the high representation of non-essential r-proteins in these datasets supports that in the absence of Dbp7, aberrant pre-60S complexes are degraded.

Interestingly, however, the essential r-protein whose presence in the Nop2-TAP particle was most strongly affected by lack of Dbp7 was uL3. uL3 is recruited to early pre-60S particles, where it plays a crucial role in stabilizing the compacted domains I and V to allow recruitment and stable incorporation of other LSU r-proteins^35,42^. Hence, it is very likely that the decreases in the amounts of eL38, uL24, uL14, eL31, and eL21 present in the Nop2-TAP particles are due to inefficient incorporation of uL3 into earlier pre-60S particles. To determine whether the catalytic activity of Dbp7 is necessary for the recruitment of uL3 to pre-60S complexes, Nop2-containing particles were isolated from the established *DBP7* complementation system. While expression of plasmid-derived Dbp7_WT_ allowed normal recruitment of uL3, Nop2-TAP particles purified from cells expressing catalytically inactive Dbp7 (Dbp7_DQGD_) contained lower amounts of uL3, similar to those purified from the Δ*dbp7* strain carry only an empty vector (Fig. 6c). Collectively, our data support a catalytic function of Dbp7 in pre-60S remodeling to facilitate release of very early AFs and snoRNPs, allowing efficient incorporation of uL3 to stabilize inter-domain interactions and downstream recruitment of AFs and r-proteins.

## Discussion

An evolutionarily conserved feature of the ribosome assembly pathway is its hierarchical nature^35,65,66^. Early pre-ribosomal complexes are characterized by their open and flexible structures, which undergo progressive compaction and stabilization during the maturation process through the formation of appropriate RNA folds and the stable incorporation of r-proteins^25,29,31,67^. Although prodigious advances in cryo-EM technology have allowed structural snapshots of an increasing number of pre-ribosomal particles, the diverse and dynamic nature of very early pre-60S complexes still renders them extremely challenging for such approaches. Accordingly, even in the yeast model system, relatively little is currently known about the maturation events taking place in early pre-60S complexes and the activities that mediate them.

The core of the mature 60S ribosomal subunit is composed of several root rRNA helices, and formation and clustering of these helices during the very early stages of subunit biogenesis is suggested to be a key licensing step for particle maturation^3,35^. Proteomic analyses of early pre-60S particles have identified several AFs, including Npa1, Npa2, Rrp5, Mak21, Noc2, and Nop4, which each contain several RNA binding motifs and are proposed to act as scaffolds during the early stages of LSU maturation^3,68–71^. Rrp5 contacts both pre-40S and pre-60S pre-rRNAs coordinating pre-ribosome assembly and interestingly, decreased pre-ribosome compaction is observed in the absence of Rrp5^40^. While Npa1 is implicated only in early pre-60S maturation, pre-rRNA contact sites in domains I, V, and VI of the 25S rRNA suggest that Npa1 also bridges long-range pre-rRNA interactions and, by tethering the 5′ and 3′ ends of the 25S rRNA in close proximity, likely contributes to stabilization of early pre-60S particles^38^. Components of the Npa1 complex are present in pre-60S particles purified via Dbp7, implying that Dbp7 also acts during the very early stages of pre-60S biogenesis when particle compaction is taking place, however, compositional analyses of the pre-60S particles isolated via these two factors suggest the action of Dbp7 downstream of the Npa1 complex. Interestingly, our data reveal that Dbp7 cross-links to the 25S-h98-99 pre-rRNA sequence also cross-linked by Npa1 and lack of Dbp7 leads to accumulation of Npa1 complex components in later pre-60S particles purified via Nop2. Together, these data suggest that Dbp7 directly or indirectly contributes to the release of the Npa1 complex from early pre-60S particles. This finding is in line with previously reported genetic interactions between Dbp7 and components of the Npa1 complex^45^.

Alongside protein scaffolds, several non-methylating snoRNPs, such as U3, U14, snR10, and snR190 tether linearly distant pre-rRNA sequences in favorable folding conformations and/or prevent formation of aberrant basepairing interactions^13,19,61^. Lack of Dbp7 or its catalytic activity lead to accumulation of snR190 in pre-60S particles, suggesting that pre-ribosome remodeling by Dbp7 contributes to the release of snR190. Interestingly, our work and that of a parallel study^61^ reveal that several other snoRNAs with modification target sites close to the identified pre-rRNA cross-linking site of Dbp7 also accumulate on pre-60S particles when the helicase is lacking. Notable among these is snR34, which is strongly accumulated on pre-ribosomes in cells lacking Dbp7 or its catalytic activity. Dbp7 is a DExD box protein and RNA helicases of this type typically mediate local strand unwinding rather than translocating along RNA substrates^54^. It is improbable, therefore, that Dbp7 directly unwinds these snoRNAs from their pre-rRNA basepairing sites. Their dissociation is likely either an indirect consequence of pre-rRNA remodeling by Dbp7 or sterically requires the physical removal of Dbp7 from pre-60S particles. CRAC analyses allow capture of transient protein–RNA interactions and mapping of protein binding sites on RNA substrates. It remains unknown, however, which parts of the protein contact the pre-rRNA/snoRNAs at the identified sites and whether any of the cross-linking sites represent interactions of the catalytic core of the helicase and therefore reflect targets of its remodeling activity. Cross-linking of Dbp7 to domain V/VI of the 25S rRNA as well as the snR190 box B region that basepairs with domain I of the 25S rRNA strongly suggests that Dbp7 interacts with pre-ribosomal complexes in which these sequences are in close proximity.

Facilitating release of snR190 and snR34 from pre-ribosomes appears to be a major function of Dbp7 as strains lacking Dbp7 display enhanced growth in the absence of these snoRNAs. Failure to release these, and potentially other, snoRNAs in the absence of Dbp7 likely represents a road-block to ongoing pre-60S maturation. A further effect of lack of Dbp7 is the reduced amounts of the so-called “A3 factors” in pre-60S particles, which is consistent with the pre-rRNA processing defects observed upon depletion of Dbp7. It is possible that the exclusion of these factors, particularly Cic1 and Nop15, may be linked to the retention of Npa1, which also binds to ITS2 close to the binding sites of these two assembly factors^38^. Available cryo-EM structures of pre-60S particles also position A3 factors on compacted domains I, II, and VI^25,29^. This suggests that proper folding of domains I, II, and VI is necessary for stable incorporation of these proteins, and therefore, that their incorporation may serve as structural checkpoint, coupling rRNA compaction to irreversible pre-rRNA processing.

Another significant consequence of lack of pre-rRNA remodeling by Dbp7 is the impaired recruitment of uL3, which positions very close to the Dbp7 pre-rRNA cross-linking sites. In mature ribosomes, this r-protein contacts both the 5′ and 3′ ends of the 25S rRNA and its recruitment is a critical milestone during pre-60S biogenesis^1,35^. Stabilization of interactions between the 5′ and 3′ ends of the LSU rRNA during early ribosome assembly is conserved from bacteria to eukaryotes, highlighting its importance for ribosome assembly. Intriguingly, the 23S rRNA of thermophilic archaea *Pyrococcus furiosus* is even circularly permutated^72^. Consistent with this, our data indicate that in the absence of Dbp7, when recruitment of uL3 is impaired, pre-60S particles are degraded, likely having not fulfilled a necessary quality control requirement. Correct positioning of uL3 is also essential for formation of the PTC and Dbp7 cross-links to helix 89, which forms part of the PTC. The enrichment of uL3 in pre-60S particles purified via Dbp7-TAP suggests the co-existence of Dbp7 and uL3 in the same pre-60S particles. In the absence of structural information on early pre-60S particles containing Dbp7 and uL3, it remains unclear whether uL3 binds this region in its final conformation while Dbp7 is still present or whether a conformational rearrangement takes place upon dissociation of Dbp7.

Together our data support a step-wise model in which tethering of domains I and VI of the 35S pre-rRNA is initially chaperoned by Npa1 and snR190 and that remodeling by Dbp7 then catalyzes an exchange of these factors for uL3 to stabilize early pre-60S particles enabling formation of the PTC and other downstream maturation events. The observation that, besides Dbp7, several other RNA helicases, including Dbp3, Dbp6, Dbp9, Has1, Mak5, Prp43, and Drs1, are present in early pre-60S particles suggests that the remodeling events induced by Dbp7 occur concurrently with other structural rearrangements catalyzed by other RNA helicases. It is likely that the concerted action of multiple helicases on different regions of early pre-60S particles is necessary to achieve accurate and efficient compaction. Structural insights into early pre-60S particles isolated via RNA helicases would likely yield further fascinating insights into the dynamics of the initial phases of pre-60S assembly and greater understanding of how the activities of different RNA helicases involved in 60S biogenesis are coordinated.

## Methods

### Molecular cloning

The *DBP7* coding sequence (CDS) was amplified from yeast genomic DNA with primers listed in Supplementary Table 3 and cloned into a pQE80-derived vector for recombinant expression of Dbp7 with an N-terminal His_10_-ZZ tag in *Escherichia coli* (*E. coli*). For generating a yeast complementation system, the *DBP7* CDS and 500 nucleotides upstream of the start codon and downstream of the stop codon was amplified from yeast genomic DNA by PCR and cloned into a pRS415 vector to allow expression from the endogenous promoter. For expression of Dbp7_DQGD_, plasmids were subjected to site-directed mutagenesis using primers to introduce a point mutation leading to substitution of glutamate E309 within the conserved DExD sequence motif with glutamine. All plasmid constructs used and generated in this study are listed in Supplementary Table 4.

### Yeast strains and growth analysis

All yeast strains generated in this study are based on *S. cerevisiae* BY4741 and are listed in Supplementary Table 5. The Δ*dbp7* yeast strain was obtained from the *S. cerevisiae* deletion collection (Euroscarf). Yeast strains expressing proteins with a C-terminal His_6_-TEV protease cleavage site-Protein A (HTP) tag, Calmodulin-binding peptide-TEV protease cleavage site-Protein A (TAP) tag, yeast-enhanced GFP Tag, 3xHA tag, or where proteins expression is from a pGAL_S_ promoter, were generated by homologous recombination using primers listed in Supplementary Table 3. Note, that the pGAL_S_-3HA cassette encodes a longer linker than the 3HA cassette used for C-terminal tagging leading to expression of proteins of slightly different lengths. A yeast complementation system was generated by transforming the Δ*dbp7* yeast strain with pRS145-derived plasmids (Supplementary Table 4) using lithium acetate–polyethylene glycol-mediated transformation. For fluorescence microscopy, a strain expressing C-terminally GFP-tagged Dbp7 from its genomic locus was transformed with plasmid for expression of Nop1-RFP. All yeast cultures were grown in Yeast Complete medium (1% yeast extract, 2% peptone/tryptone, 2% glucose or galactose) or synthetic medium lacking one amino acid (1.9 g/L yeast nitrogen base without amino acid (Formedium), 5 g/L ammonium sulfate, 2% dextrose, and corresponding amount of complete supplemented dropout of -HIS/-LEU/-URA (Formedium)). For all experiments, cultures were propagated for >12 h in exponential phase before harvesting. Cell growth was monitored every 90 or 120 min in liquid culture or through spotting of ten-fold serial dilutions of cultures on agar medium followed by examination after 2 or 3 days. For transient depletion of Dbp7, the pGAL_S_-3HA-*DBP7* yeast strain was grown exponentially in medium containing 2% galactose and then subsequently in media containing 2% glucose for 12 h before harvesting. The *snR190*_*mut*_ strain lacking snR190 was constructed through genome editing using the CRISPR-Cas9 approach^73^. As snR190 is expressed from a dicistronic gene also encoding the essential U14 snoRNA, inhibition of snR190 expression was achieved by introducing mutations in the K-loop motif of snR190 (box C and adjacent stem) to destabilize the snoRNA post-transcriptionally. A pML104-based vector (URA3 marker) for expression of Cas9 was engineered to express a guide RNA targeting Cas9 to the *SNR190* genomic locus, by inserting the annealed oligonucleotides OHA494 and OHA495 into pML104 previously linearized by BclI and SwaI. The resulting plasmid was co-transformed into BY4741 strain along with a donor single-stranded oligonucleotide (OHA496) containing the mutations to be introduced. Transformants were selected on synthetic medium lacking uracil and the pML104-based plasmid was rapidly counter-selected on plates containing 5-fluoroorotic acid. To validate the presence and specificity of the mutations, the *SNR190* locus was PCR-amplified from genomic DNA of selected clones using OHA497 and OHA498 primers, and sequenced.

### RNA isolation and northern blotting

RNA was extracted from yeast cells, sucrose density gradient fractions, and pre-60S particles immobilized on IgG Sepharose using phenol-chloroform-based extraction^47,49,74^. For analysis of pre-rRNAs, 6 μg of total RNA or RNA isolated from pre-60S particles were separated on a denaturing (glyoxal) 1.2% ultrapure agarose gel and transferred by vacuum blotting onto a nylon membrane. For analysis of snoRNAs, RNAs were separated on 8% denaturing (7 M Urea) polyacrylamide gels. RNA targets were detected using 5′-[^32^P]-labeled antisense DNA oligonucleotides listed in Supplementary Table 3. Membranes were exposed to phosphorimager screens and radioactive signals were detected using a Typhoon FLA 9500 (GE Healthcare Limited) phosphorimager. Images were quantified using ImageStudio™ Lite (LI-COR Biosciences) or ImageQuant (GE Healthcare Limited) software.

### Isolation of pre-ribosomal particles

Complexes associated with TAP-tagged proteins were isolated from appropriate yeast strains^49^. Cells were lysed by grinding in liquid nitrogen in a buffer containing 50 mM Tris-HCl pH 7.8, 150 mM NaCl, 1.5 mM MgCl_2_, 0.05% NP-40, 2 mM DTT and protease inhibitors. Clarified lysates were incubated with IgG sepharose beads for 2 h to capture protein A-tagged proteins. After subsequent washing, bound complexes were eluted with TEV protease overnight at 4 °C. Proteins in the input and eluate were then precipitated using 25% TCA, separated by SDS-PAGE, and analyzed by western blotting using the antibodies listed in Supplementary Table 6 or by MS. For analysis of co-purified RNAs, RNAs were extracted and detected by northern blotting as described above.

### Proteomics analysis and data processing

Isolated pre-ribosomal proteins were separated by SDS-PAGE, and each lane was cut into 21 slices. All gel slices were reduced, alkylated, and then digested with modified trypsin. The resulting peptides were extracted from the gel and vacuum-dried.

Dried peptides were dissolved in 2% acetonitrile (ACN) containing 0.1% formic acid (FA) and analyzed using a Q Exactive HF mass spectrometer (ThermoFisher Scientific) coupled with an Ultimate 3000 RSLC system (Dionex). Peptides were loaded on a reverse-phase C18 pre-column (Dionex, 5-mm long, 0.3 mm inner diameter), and desalted for 3 min using buffer A (0.1% FA). After 3 min, the pre-column was switched online with a self-made analytical column (30-cm long, 75 μm inner diameter, packed with 1.9 μm ReproSil-Pur C18-AQ beads (Dr. Maisch GmbH)). Trapped peptides were separated with a linear gradient of 5–45% buffer B (80% FA and 0.1% FA) at a flow rate of 300 nl/min. The total run time was 58 min. Both the pre-column and the column were maintained at 50 °C. The Q Exactive HF was operated in a data-dependent acquisition manner where one full MS scan across the 350–1600 *m*/*z* range was acquired at a resolution setting of 60,000 FWHM (full width, half maximum) to select up to 30 most abundant peptide precursors. Precursors were fragmented by higher collision energy dissociation (HCD) with nitrogen at a normalized collision energy setting of 28%, and their production spectra were recorded at resolution of 15,000 FWHM with the maximum ion injection time of 60 ms.

The MS raw files were processed by MaxQuant (version 1.6.5.0)^75^ and MS/MS spectra were searched against UniProt *S. Cerevisiae* database (downloaded on Feb 2019 with 9731 entries) using default settings. Trypsin was used for protein digestion with up to two mis-cleavages. Methionine oxidation and cysteine carbamidomethylation were defined as variable and fixed modifications, respectively. The false discovery rate (FDR) was set to 1% for both peptide and protein identifications. Subsequent data analysis was conducted with Perseus (version 1.6.2.3)^76^. After removing all decoy hits and potential contaminant entries, proteins identified only with modified peptides were filtered out. The intensity-based absolute quantification (iBAQ^77^) values of the remaining proteins were log2-transformed and median normalized. Subsequently, the iBAQ differences between input and eluate samples were calculated without any imputation.

### Sucrose density gradient centrifugation

Exponentially growing cells were lysed by grinding in liquid nitrogen in a buffer containing 50 mM Tris-HCl pH 7.8, 150 mM NaCl, 1.5 mM MgCl_2_, 0.05% NP-40, 2 mM DTT, and protease inhibitors. Cleared whole-cell lysates were separated on 10–45% sucrose density gradients in an SW-40Ti rotor for 16 h at 23,500 rpm^78^. Gradients were fractionated and the absorbance of each fraction at 260 nm was measured using a NanoDrop™ (Thermo Scientific). Proteins in each fraction were precipitated with 20% TCA and analyzed by western blotting. RNAs present in each fraction were extracted and analyzed by northern blotting.

### RiboMeth-seq

Yeast strains were grown exponentially to an OD_600_ of 0.8. Total RNA was extracted and 5 μg was used for RiboMeth-seq^55^. In brief, 5 µg total RNA was fragmented under denaturing conditions in alkaline buffer (pH 9.9). Then, the RNA was separated on a denaturing (urea) polyacrylamide gel, fragments in the size range 20–40 nt were excised and ligated to adapters using a modified tRNA ligase. cDNA was made using Superscript III (Thermo Fisher Scientific) and sequenced on a PI Chip v3 using the Ion Proton platform. Reads were mapped to the yeast transcriptome, and the RMS score in rRNAs (fraction methylated) calculated as “score C”^55^. In a few cases, a barcode correction was applied when calculating the RMS score^56^. The numbers of sequencing reads mapping to specific RNAs was used as a measure of their levels and is presented as reads per kilobase of transcript, per million mapped reads (RPKM) ± SEM. Statistical significance was determined using two-tailed Student’s *t*-test (*p* < 0.05).

### Cross-linking and analysis of cDNA (CRAC)

For cross-linking and analysis of cDNA (CRAC) experiments^57–59,79^, wild-type yeast and a yeast strain expressing C-terminally HTP-tagged Dbp7 from its genomic locus were first grown exponentially in low uracil medium (10 mg/L uracil) supplemented with 100 μM 4-thiouracil until OD_600_ of 0.5 was reached. 4-thiouracil was then added to a concentration of 1 mM and cultures were grown for an additional 4 h prior to harvesting and cross-linking at 365 nm using two rounds of 600 mJ/cm^2^ in a Stratalinker (Agilent Technologies). Protein–RNA complexes were subsequently isolated on IgG sepharose and NiNTA. RNAs were trimmed by partial RNase digestion and the RNA fragments were 5′ [^32^P]-labeled and ligated to adapters. cDNA libraries were prepared for Illumina deep sequencing by reverse transcription and PCR amplification. Sequencing reads were processed and subjected to quality control, and after mapping to the *S. cerevisiae* genome (S228C) using Bowtie 2 (version 2.2.4) only reads containing a specific T-C mutation were retained. The distribution of reads derived from different RNA species was determined using pyCRAC read counting^80^. Custom python scripts (Supplementary Methods) were used to depict the numbers of reads mapping to each nucleotide of the 25S rRNA on the available secondary structures of the rRNA^60^ and tertiary structure of the State C pre-60S particle using a color scale^81^. Dbp7-HTP cross-linking sites were visualized on the pre-60S structure using PyMOL.

### Purification of recombinant proteins and in vitro ATPase assays

His-ZZ-tagged recombinant proteins were expressed in *E. coli* (BL21 Codon Plus) with 1 mM ITPG overnight at 18 °C, cells were pelleted, resuspended in lysis buffer (50 mM Tris-HCl pH 7, 500 mM NaCl, 1 mM MgCl_2_ 10 mM imidazole, 1 mM PMSF, 10% glycerol), and lysed by sonication. Cleared cell lysate was supplemented with polyethyleneimine to a final concentration of 0.05% to precipitate protein-associated nucleic acids. The resulting soluble fraction was then incubated with cOmplete His-Tag purification resin (Roche). Following thorough washing steps, first with low and then high salt buffer (50 mM Tris-HCl pH 7.0, 500/1000 mM NaCl, 1 mM MgCl_2_, 30 mM imidazole, 10% glycerol), bound proteins were eluted with a buffer containing 50 mM Tris-HCl pH 7, 500 mM NaCl, 1 mM MgCl_2_, 300 mM imidazole, and 10% glycerol. Fractions containing proteins were pooled and dialyzed against a buffer containing 50 mM Tris-HCl pH 7, 120 mM NaCl, 2 mM MgCl_2_, 20% glycerol. The quantity of the purified protein was measured using a Bradford assay.

For in vitro NADH-coupled ATPase assays^82,83^, reactions were carried out in solution containing 50 mM Tris-HCl pH 7.4, 25 mM NaCl, 2 mM MgCl_2_, 4 mM ATP, 1.5 mM PEP (phosphoenolpyruvate), 450 μM NADH, 1.5 μM recombinantly expressed and purified RNA helicase, and 1.5 μM RNA (5′-GUAAUGAAAGUCCAUGUAAAACAAAACAAAAC-3′). Absorbance at 340 nm was measured every 50 s for 30 min at 30 °C using a Gen5 Microplate Reader (Biotek) and the rate of ATP hydrolysis was calculated using Eq. (1).

1 $${nM}\,{ATP}\,{hydrolysed}\times {{{{{{{\mathrm{sec}}}}}} }}^{-1}=-\frac{d{A}_{340}}{{dt}}\times {K}_{{path}}^{-1}\times {10}^{6}$$

### Reporting summary

Further information on research design is available in the Nature Research Reporting Summary linked to this article.

## Supplementary information

Supplementary Information

Reporting summary

## Data Availability

The CRAC datasets and their analyses for Dbp7-HTP and the wild-type yeast control shown in Fig. 4 are deposited in Gene Expression Omnibus (GEO) database [http://www.ncbi.nlm.nih.gov/geo/] under the accession code GSE160734. Sequencing reads were mapped to the *S. cerevisiae* genome (https://www.yeastgenome.org/strain/s288c). The RMS datasets presented here in Fig. 2a, b are also deposited in the GEO data base under the accession code GSE161347. The mass spectrometry proteomics data underlying Fig. 1g have been deposited to the ProteomeXchange Consortium via the PRIDE partner repository [https://www.ebi.ac.uk/pride/] with the dataset identifier PXD022625. MS-MS spectra were searched against the UniProt *S. Cerevisiae* database (https://www.uniprot.org/proteomes/UP000002311; downloaded on Feb 2019 with 9731 entries). Source data are provided with this paper.

## Source data

Source Data

## Code availability

Custom python scripts used for visualizing the number of reads in the Dbp7-HTP CRAC dataset mapping to each nucleotide on secondary and tertiary structures of the 25S rRNA are available as Supplementary Methods.
