# Supplementary material for: The RNA helicase Dbp7 promotes domain V/VI compaction and stabilization of inter-domain interactions during early 60S assembly
Source: Nat Commun. 2021 Oct 22;12:6152. doi: 10.1038/s41467-021-26208-9 (PMC8536713; doi:10.1038/s41467-021-26208-9)
Supplement: Supplementary file 1 — Supplementary Information [file 41467_2021_26208_MOESM1_ESM.pdf]

Supplementary Information

The RNA helicase Dbp7 promotes domain V/VI compaction and stabilization of inter-domain interactions during early 60S assembly

Gerald Ryan R. Aquino, Philipp Hackert, Nicolai Krogh, Kuan-Ting Pan, Mariam Jaafar, Anthony K. Henras, Henrik Nielsen, Henning Urlaub, Katherine E. Bohnsack and Markus T. Bohnsack

Supplementary Figures

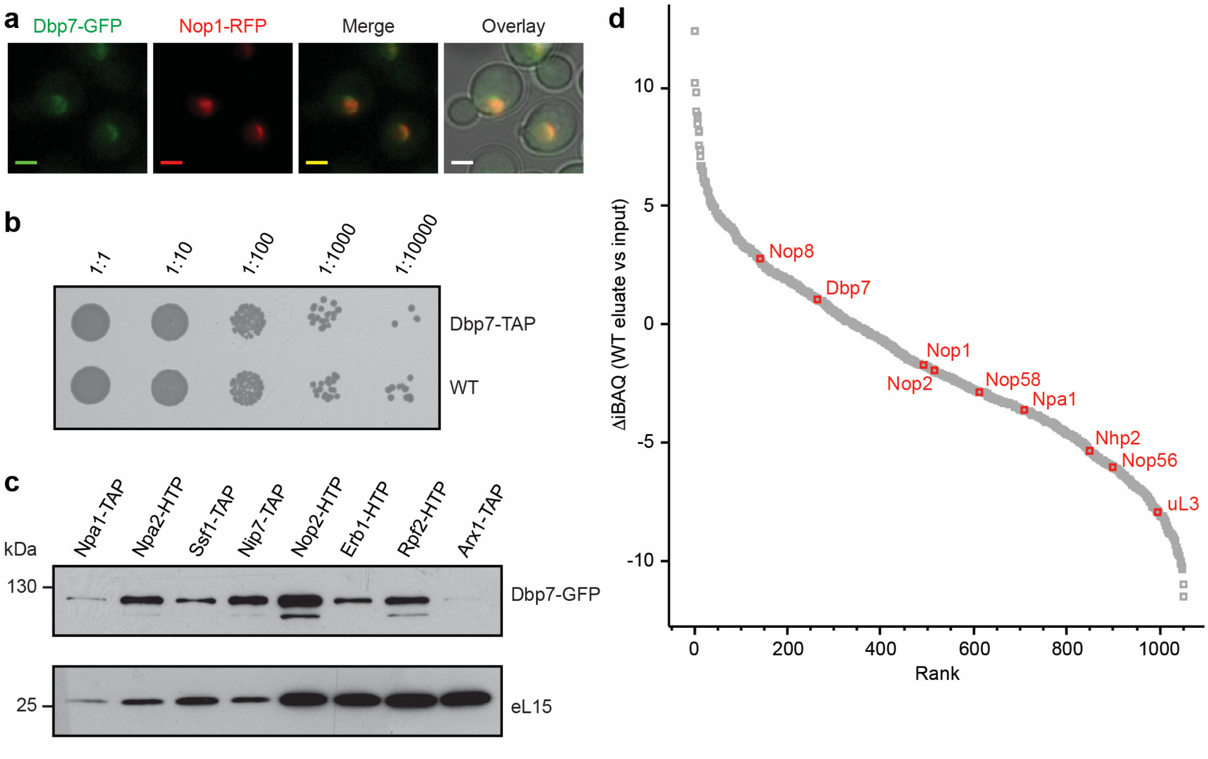

Supplementary Fig. 1. Dbp7 is a nucleolar protein required for normal growth. (a) Cells expressing Dbp7 with a C-terminal GFP tag were visualized using a fluorescence microscope. Nop1-RFP was used as nucleolar marker. Fluorescence microscopy images were overlaid with a bright field image. Co-localization appears in yellow. Scale bar = 2 μm. n= >200 cells imaged in one biological replicate. (b) Equal numbers cells from exponentially growing cultures of the wild-type (WT) and Dbp7-TAP strains were serially diluted and spotted onto a plate. Growth was documented after 48 h of incubation at 30 °C. (c) Whole cell lysates from strains expressing Dbp7-GFP and TAP/HTP-tagged pre-60S AFs were used for pulldown assays. Proteins in eluates were detected by western blotting using anti-GFP and anti-eL15 antibodies. Data from a single experiment is shown. (d) Proteins in input and eluate samples of pulldowns shown in Fig. 1f,g were analyzed by mass spectrometry. Ranked differences in intensity-based absolute quantification (iBAQ) values between the input and eluate of the WT sample are shown.

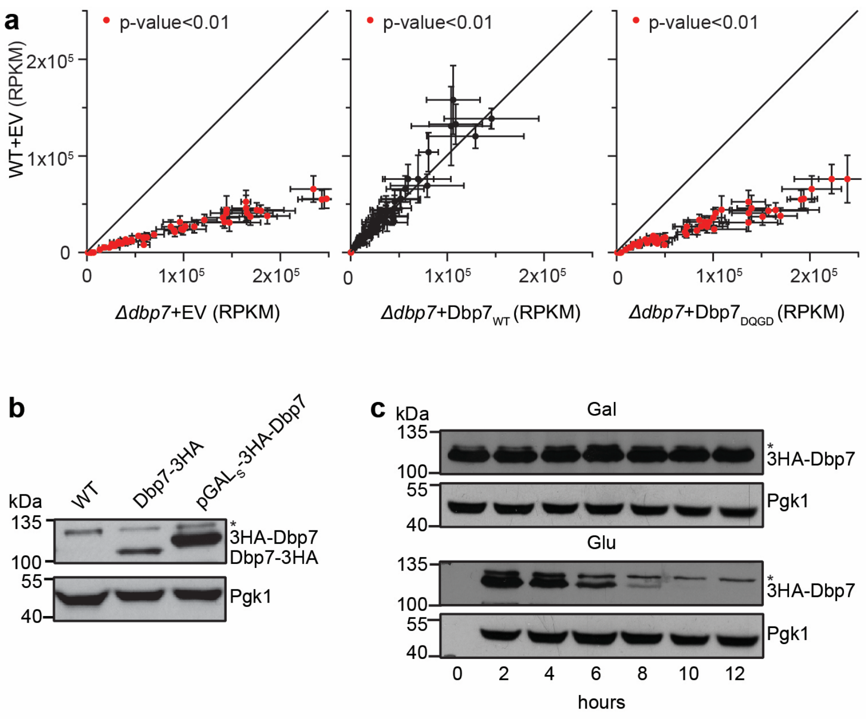

Supplementary Fig. 2. Relative tRNA expression in cells lacking Dbp7 or its catalytic activity and expression of Dbp7 from endogenous and pGal$_s$ promoter. (a) The normalized (Reads Per Kilobase of transcript, per Million mapped reads; RPKM) numbers of sequencing reads mapping to tRNAs was determined in the RMS datasets for wild-type yeast containing an empty vector (EV) and the *Δdbp7* strain complemented with EV or plasmids for expression of Dbp7$_{WT}$ or Dbp7$_{DQGD}$. Relative tRNA levels from n=3 biologically independent RMS experiments are shown as mean ± standard deviation (see Source data for individual data points). (b) Total protein was extracted from two biological replicates of wild-type yeast (WT) cells expressing Dbp7-3HA from the endogenous *DBP7* promoter and cells expressing 3HA-Dbp7 from a pGAL$_S$ promoter and protein levels were analyzed by western blotting using antibodies against the HA tag and Pgk1. Asterisk indicates a non-specific interaction of the anti-HA antibody. (c) Cells from the pGAL$_S$-3HA-Dbp7 strain were grown exponentially for 12 h in YP medium containing galactose (Gal) or glucose (Glu), and Dbp7 expression, monitored every two hours, was analyzed as in (b). Asterisk indicates a non-specific interaction of the anti-HA antibody. Data from a single experiment is presented.

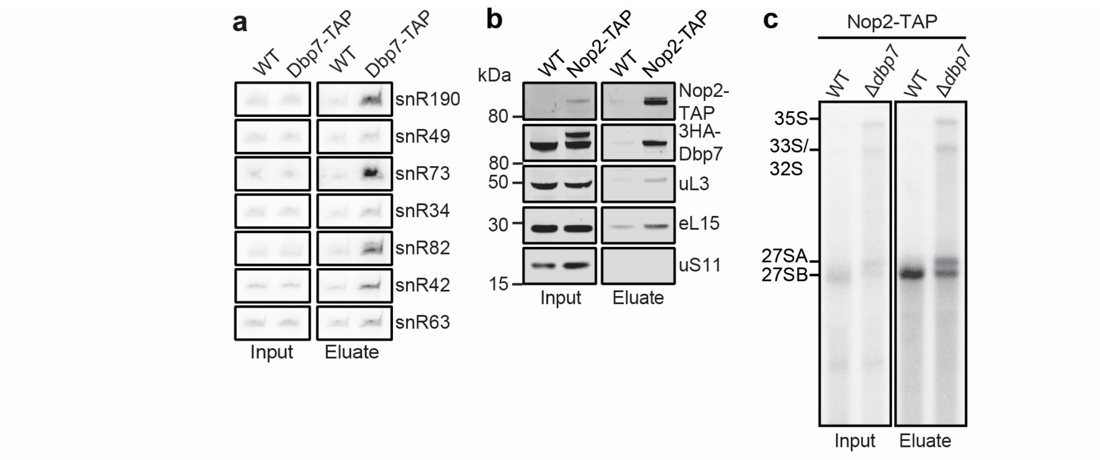

Supplementary Fig. 3. Analysis of snoRNA and pre-rRNA compositions of pre-60S particles isolated via Dbp7-TAP or Nop2-TAP. (a) Extracts from wild-type yeast or a strain expressing TAP-tagged Dbp7 were used for pulldown assays on IgG sepharose and co-purified RNAs were extracted. After separation by denaturing PAGE, the indicated snoRNAs were detected by northern blotting analysis using probes hybridizing to each snoRNA. Two experiments were performed and representative data are shown. (b) Extracts from cells expressing 3HA-Dbp7 in a wild-type background (WT) or in a strain expressing Nop2-TAP were used for pulldown assays on IgG sepharose. Input (0.5%) and eluate were separated by denaturing PAGE and analyzed by western blotting using antibodies against the HA tag (Dbp7), the CBP tag (Nop2), and endogenous uL3, eL15 and uS11. Data from one experiment are shown. (c) Pulldowns were performed as in (b) and RNAs present in input samples (0.2%) and eluates were extracted. RNAs were separated by denaturing agarose gel electrophoresis and pre-rRNA species were detected by northern blotting. Data from one experiment are shown.

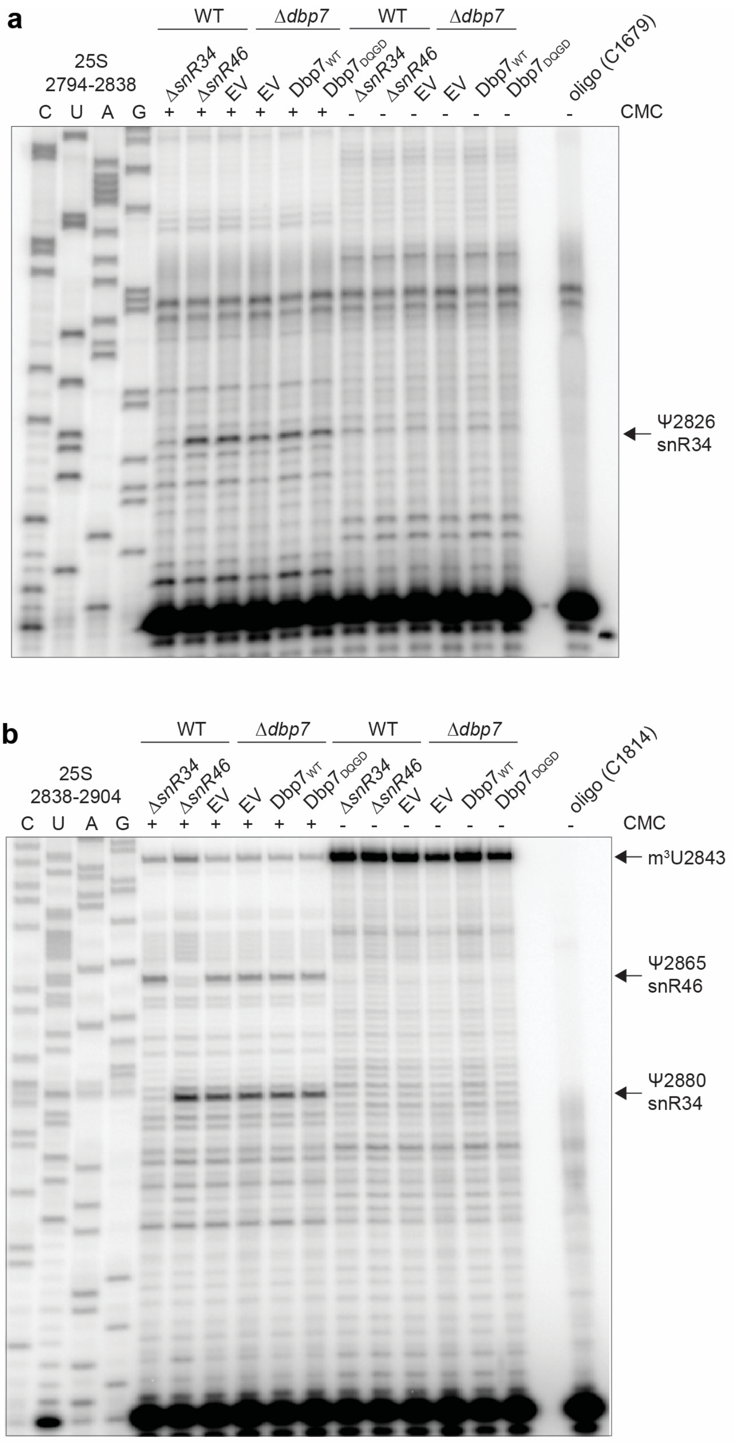

Supplementary Fig. 4. snR34- or snR46-guided pseudouridylation of the 25S rRNA is not affected by lack of Dbp7. (a,b) Total RNA from the indicated yeast strains was labelled with CMC and primer extension was performed using a radiolabeled primer basepairing to 25S rRNA nucleotides 2794-2838 (a) or 2838-2904 (b). cDNA fragments were separated by

denaturing gel electrophoresis alongside a sequencing ladder and radiolabeled oligonucleotides were detected using a phosphorimager. Prominent primer extension stops arising due to the presence of modified RNA nucleotides are indicated on the right. Data from a single experiment are shown in (a) and (b).

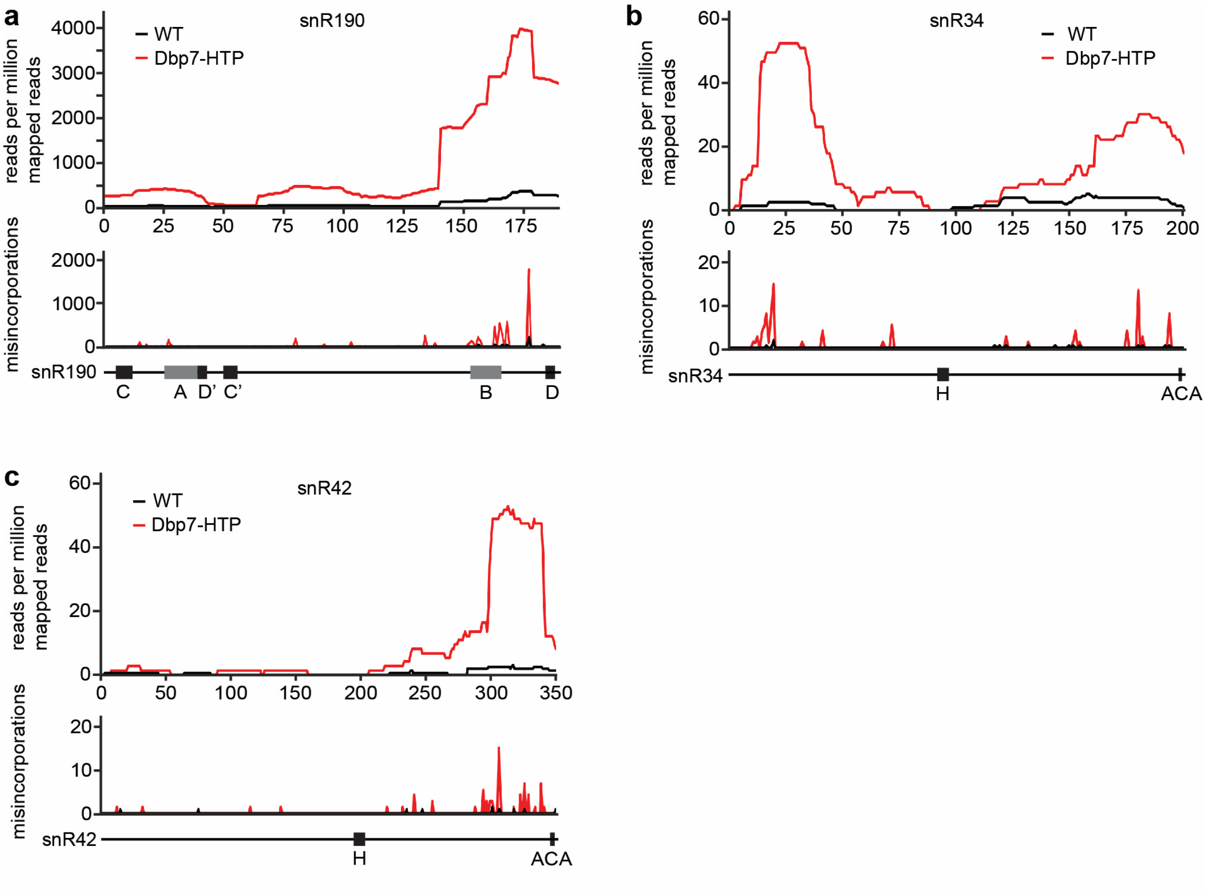

Supplementary Fig. 5. Dbp7-HTP crosslinking profiles on selected snoRNAs. (a-c) The number of sequencing reads mapping to each nucleotide of *snR190* (a), *snR34* (b) and *snR42* (c) genes in representative Dbp7-HTP and wild-type PAR-CRAC data set are shown. The normalized number of T-C misincorporations corresponding to each nucleotide are presented in the lower graph. Schematic representations of the snoRNAs are shown below with the positions of evolutionarily conserved sequences (boxes; A, B, C, C', D, D', H and ACA) highlighted with black and grey rectangles.

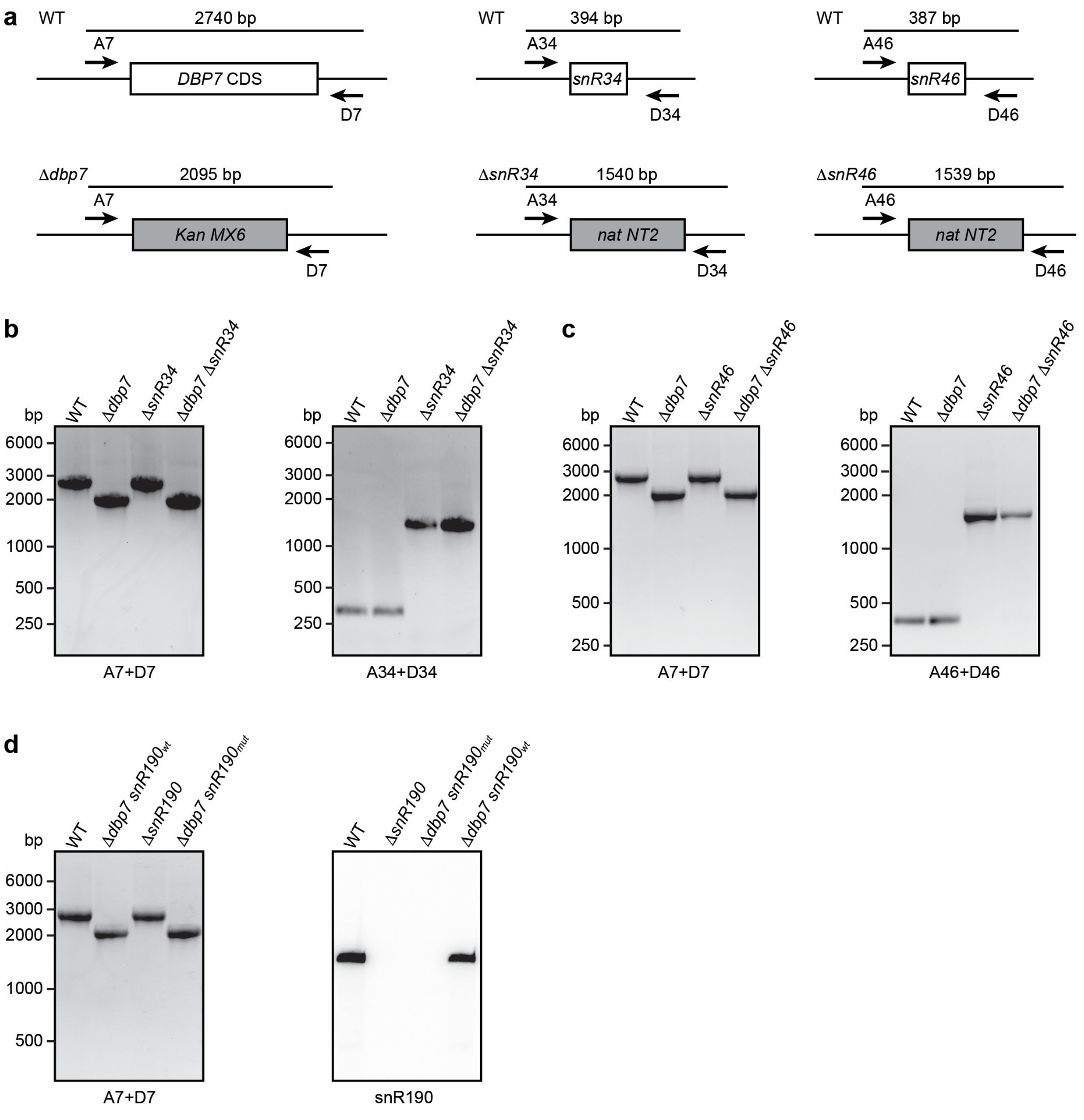

Supplementary Fig. 6. Verification of yeast strains lacking *dbp7* or selected snoRNA genes, or carrying point mutations in *snR190*. (a) Schematic view of the PCR-based approach to verify the identity of the yeast strains generated. (b-d) Genomic DNA extracted from the indicated yeast strains was used as a template for PCR reactions using the primer pairs spanning the *DBP7* locus (A7+D7; b, c, d left panel), *SNR34* locus (A34+D34; b right panel) or the *SNR46* locus (A46+D46; c right panel). PCR products were separated by agarose gel electrophoresis and visualized using SafeView and UV light. (d, right panel) Total RNAs extracted from the wild-type (WT), *snR190*$_{mut}$, Δ*dbp7 snR190*$_{wt}$, or Δ*dbp7 snR190*$_{mut}$

strains were separated by denaturing PAGE and snR190 was detected by northern blotting. The identities of the strains were verified in one experiment.

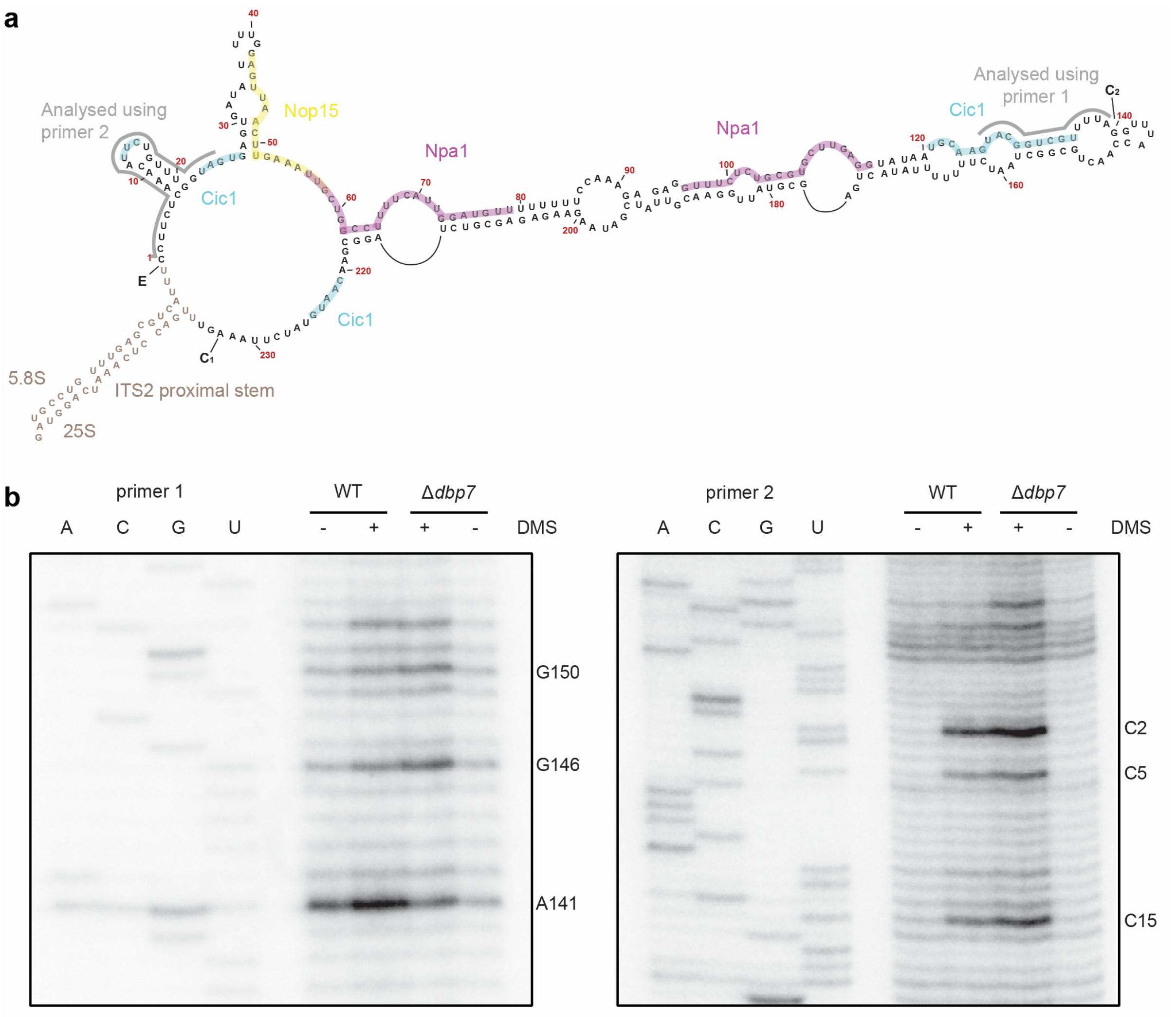

Supplementary Fig. 7. Structural analysis of ITS2 region via DMS structure probing. (a) Secondary structure of the hairpin loop model of ITS2. Highlighted in blue, yellow and purple are the binding site of assembly factors Cic1, Nop15, and Npa1 respectively. The regions analyzed in (b) are indicated by grey lines. (b) Nop2-containing particles from WT and Δ*dbp7* cells were immobilized on IgG sepharose and treated with DMS (+) or left untreated (-). RNAs associated with the complex were extracted and used as template for primer extension using two different radiolabeled primers (primer 1 and primer 2). cDNA transcripts were separated by denaturing PAGE alongside a sequencing ladder. Signals were visualized using a phosphorimager. Data from one experiment are shown.

Supplementary Tables

Supplementary Table 1. Growth rates of yeast strains used in this study.

Name	Genotype	Mean doubling time (h)
YMB006/BY4741a	MATa;hisΔ1;leu2Δ0;met15Δ0;uraΔ0	1.67
YMB1470	YMB006; *dbp7*::kanMX6	3.18
YMB1693	YMB006; *dbp7*-TAP (kanMX6)	1.73
YMB1541 (Gal)	YMB006; pGAL$_S$-3xHA-*dbp7* (*n*atNT2)	2.21
YMB1541 (Glu)	YMB006; pGAL$_S$-3xHA-*dbp7* (*n*atNT2)	2.17
YMB1974	YMB006; *snr34*::natNT2	1.68
YMB1976	YMB006; *snr46*::natNT2	1.64
YMB1978	YMB1470; *snr34*::natNT2	2.90
YMB1979	YMB1470; *snr46*::natNT2	3.17
YMB1985	BY4741	1.58
YMB1987	*snr190*mut	1.76
YMB1988	*dbp7*::kanMX6 *snr190*mut	2.81
YMB1989	*dbp7*::kanMX6_*snr190*wt	3.22

Supplementary Table 2. Changes in protein composition of pre-60S particles enriched with Nop2-TAP from cells expressing and lacking Dbp7.

Ribosomal Protein	Fold-change (WT vs Δ*dbp7;* Log$_2$ of fold-change)
uL3	-0.5
uL4	-0.5
eL6	0.1
uL30	0.3
eL8	0.1
uL6	-0.3
eL14	0.1
eL15	0.5
uL13	0.3
uL22	0.2
eL18	0.4
eL19	0.4
eL20	-0.1
eL21	-0.8
eL22	0
uL14	-1.3
uL23	-0.3
uL24	-1.5
eL27	-0.2
uL15	-0.8
eL30	0
eL31	-1.2
eL32	-0.3
eL33	-0.1
eL34	0.4
uL29	-0.5

eL36	-0.1
eL37	-0.4
eL38	-3.0
Assembly Factors	
Brx1	0.1
Cbf5	1.54
Cic1	-1.1
Dbp10	0.0
Dbp2	0.2
Dbp6	0.6
Dbp9	-1.3
Drs1	-1.6
Ebp2	-0.5
Erb1	0.1
Gar1	2.16
Has1	-0.6
Loc1	0.1
Mak5	1.1
Mak11	1.6
Mak16	-1.0
Mak21	0.6
Mrt4	-1.5
Nip7	0.1
Noc3	-2.1
Nog1	-1.5
Nop2	0.0
Nop4	1.16
Nop7	-1.2
Nop8	2.0
Nop12	2.2
Nop15	-1.0
Nop16	-0.5
Npa1	1.1
Npa2	2.7
Nsa1	-0.8
Nsa2	-1.6
Nug1	-1.9
Prp43	1.7
Rlp24	-0.5
Rlp7	-2.6
Rpf1	-0.1
Rrp1	0.3
Rrp5	0.6
Rrp14	-0.8
Rrp15	-1.1
Rrs1	0.6
Rsa3	0.4
Spb1	-1.6
Spb4	-0.7
Ssf1	-0.7
Ssf2	-0.3
Ytm1	-1.3

Supplementary Table 3. DNA oligonucleotides used in this study.

Name	Sequence (5'-3')	Application
oMB1462	CGGTTTTAATTGTCCTA	Northern blot probe for ITS1
oMB1468	TGAGAAGGAAATGACGCT	Northern blot probe for ITS2
oMB2213	GTCACAGGCGAAATATCATCAAAGTTAATC	Northern blot probe for snR73
oMB2543	ATCCCGGCCGCCTCCATCAC	Northern blot probe for Scr1
oMB6604	ATATATAGATCTGCTATAGCGCTGTTTGTTCCGTTCTC	Molecular cloning for expression in yeast ($DBP7_{WT\_500updown}$)
oMB6605	ATATATGCGGCCGCCGTTGTTAGCATGAATATAG	Molecular cloning for expression in yeast ($DBP7_{WT\_500updown}$)
oMB6606	GCCTGAAAAATGAACAGAGTTTTTA	Checking genomic deletion (Dbp7-A)
oMB6609	CAACAGAATAGAAAAACGGAAGAAA	Checking genomic deletion (Dbp7-D)
oMB6610	CTAAGGTATATCGTATTGGATCAAGGAGATAAGTTAATGGAATTGG	Site-directed mutagenesis ($Dbp7_{DQGD}$)
oMB6611	CCAATTCCATTAACTTATCTCCTTGATCCAATACGATATACCTTAG	Site-directed mutagenesis ($Dbp7_{DQGD}$)
oMB6620	ATATATAGATCTATGAGCGATGAAGATTCTATGCTG	Molecular cloning for recombinant expression
oMB6621	ATATATGCTAGCGTAGTTAAACTCACTTGCTATTTG	Molecular cloning for recombinant expression
oMB6691	GTTTCGTATGGCTCGTATGGCAGAGAAGCAAATAGCAAGTGAGTTTAACTACCGGATCCCCGGGTTAATTAA	3xHA genomic tagging (Dbp7-Fw)
oMB6692	CTTTGTCTTCATAGTATACAATTTTTTTTTATATGAATTAATGCTTGTTCTTGTCTATGAATTCGAGCTCGTTTAAAC	3xHA genomic tagging (Dbp7-Rev)
oMB6750	TATTCATATTCCAAATAAGCATACTTATTCAGCAATTTAACACCAAGATGCGTACGCTGCAGGTCGAC	pGALs-3xHA genomic tagging (Dbp7-Fw)
oMB6751	GCAGTGTCCTCATTGGTAGTGAAGTTTAACAGCATAGAATCTTCATCGCTCATCGATGAATTCTCTGTCG	pGALs-3xHA genomic tagging (Dbp7-Rev)
oMB6888	CGTGCGTCTGATTATGGTCC	Northern blot probe for snR63
oMB7265	GTTTCGTATGGCTCGTATGGCAGAGAAGCAAATAGCAAGTGAGTTTAACTACCGTACGCTGCAGGTCGAC	TAP/GFP genomic tagging (Dbp7-Fw)
oMB7266	CTTTGTCTTCATAGTATACAATTTTTTTTTATATGAATTAATGCTTGTTCTTGTCTATTTAATCGATGAATTCGAGCTCG	TAP/GFP genomic tagging (Dbp7-Rev)
oMB8024	CAATCTTTTGAAAAAGGGTGTCAATCCAAAAGCTAAAAGACCTTCTAACGAAAAAATGGAAAAGAGAAGATGGAAAAAGAATTTC	TAP genomic tagging (Nop2-Fw)

oMB8025	AGATTAGAGAGAGAAAACTATGCTAACATGATGCCA CTACGTTTGTGGGAACTACGACTCACTATAGGGCGA ATTGGG	TAP genomic tagging (Nop2-Rev)
oMB8028	ATATTTCAAAGCACTTTACCTCCAATACAAAAAGGTT GGTAAATGGCGCGAAGATATGGAAAAGAGAAGATG GAAAAAGAATTT	TAP genomic tagging (Urb2-Fw)
oMB8029	TGTTATTAAACGTGAGCAGAGAAATGCCTTTTGAAAA CACACTAAAACACATAAGTACGACTCACTATAGGGC GAATTGGG	TAP genomic tagging (Urb2-Rev)
oMB8097	GAAGACGATAAGTACATTGAAAAGTCTGTGAAAAAC AATCTTTTGAAAAAGGGTGTCAATCCAAAAGC	TAP genomic tagging (Nop2-Fw2)
oMB8098	TGATATATATATATATATATATATATGTAGACAGAGAA AGATTAGAGAGAGAAAACTATGCTAAC	TAP genomic tagging (Nop2-Rev2)
oMB8101	CAATTGAACGCTTTCCTTGACACACCTGGGAAACAA TATTTCAAAGCACTTTACCTCCAATAC	TAP genomic tagging (Urb2-Fw2)
oMB8102	TTTATCAATTTTTACTTGTTTAAGCTCCGTCACCCTG TTATTAAACGTGAGCAGAGAAATGCC	TAP genomic tagging (Urb2-Rev2)
oMB8426	CGCTCTCTTCTTATCGATAACG	DMS structure probing of ITS2
oMB8427	GCACGCAGAGAAACCTCTCTTTGG	DMS structure probing of ITS2
oMB8945	CCTTGTCGTCATGGTCGAATCG	Northern blot probe for snR190
oMB8946	CTCCCGGTAACCAGGCCAGCAG	Northern blot probe for snR82
oMB8985	CGGACTTCCTATCCTGTCC	Northern blot probe for snR34
oMB8986	GCAACCTCTAACGGCGATGG	Northern blot probe for snR42
oMB9023	CGGGATTCGTTTACCATAGGCTACC	Northern blot probe for snR49
oMB9046	CTTCCCTTTGGAAATCGGAAATTC	Northern blot probe for snR46
oMB9050	TAGCGAGCTTGAAAAAGAATCTAGCGAGTCAGAAGC TGTCAAGAAGGCTAAAAGTCGTACGCTGCAGGTCGA C	GFP genomic tagging (Cic1-Fw)
oMB9051	TTTTTCTTCACAAGAAAAAAATGAGAGAAAAGATAGA TAAGGAGGAAACAAATTAATCGATGAATTCGAGCTC G	GFP genomic tagging (Cic1-Rev)
oMB9491	CAACAACAATTTTGAGTATATTTACCACATTTTTTCTG ATTTTTTTTTAAGAGGGTCGTACGCTGCAGGTCGAC	Genomic deletion (snR34-Fw)
oMB9492	GAAACTGATCTTCAAGACTTTTTACCTGCAAGAGACA GTACGTAAATATATGAGATCGATGAATTCGAGCTCG	Genomic deletion (snR34-Rev)
oMB9493	GGTTGTATAAAAAGGCAGGACTTCG	Checking genomic deletion (snR34-A)
oMB9494	CATACCTCGGACTAAGTACAGAAAC	Checking genomic deletion (snR34-D)
oMB9511	TGTAAATGTTAGTGAAAGTTTTAGGGTTCTTGTGACA AACTTCACCAACATAAACCGTACGCTGCAGGTCGAC	Genomic deletion (snR46-Fw)
oMB9512	GCATTTTTAGAAGGCTTAATCGATATCAGAACTACCA ATTGTTCCTACTACAAGAAATCGATGAATTCGAGCTC G	Genomic deletion (snR46-Rev)
oMB9513	CCTTTATTGTCATGGGTCAAGAC	Checking genomic deletion (snR46-A)

oMB9514	CGACCAGCTCTTTAGCATCCATAGCTAC	Checking genomic deletion (snR46-D)
C1679	GCAATGTCGCTATGAACGCT	Primer extension (25S-Ψ2826)
C1814	ATCCAACGCTTACCGAAT	Primer extension (25S-Ψ2865 and Ψ2880)
C1816	TGAATACAAACCATGAAAGTGT	PCR for sequencing ladder template
OHA494	GATCAAGAGACACCATTATCATCAGTTTTAGAGCTA G	CRISPR-Cas9, cloning into pML104
OHA495	CTAGCTCTAAAACTGATGATAATGGTGTCTCTT	CRISPR-Cas9, cloning into pML104
OHA496	GAAATTTCATGACACTTTTTAAACAGAAAAGAAGAA TTGATTGGAACTATTGATAATGGTGTCTCTTCTTTCC TCGTCCGATTCGACCATGACGACAA	CRISPR-Cas9: donor template for mutagenesis
OHA497	CGACTTCAGCATTGCACTCC	Genomic amplification of SNR190 locus
OHA498	CCGAGAGTACTAACGATGGG	Genomic amplification of SNR190 locus

Supplementary Table 4. Constructs used in this study.

Name	Description	Application
pMB31	pRS415	Protein expression in yeast (complementation)
pMB203	pFA6a-3xHA-His3MX6[1]	Amplification of 3xHA cassette
pMB318	pQE-80-nHis10-ZZ-TEV-Dbp7	Recombinant protein expression
pMB541	pUN100 mRFP-Nop1	Immunofluorescence microscopy
pMB938	pFA6a-natNT2[2]	Amplification of natNT2 cassette
pMB939	pYM13[2]	Amplification of TAP cassette
pMB942	pYM16[2]	Amplification of 6xHA cassette
pMB951	pYM25[2]	Amplification of GFP cassette
pMB1005	pYM-N32[2]	Amplification of pGALs-3HA cassette
pMB1364	pRS415-DBP7$_{WT_500updown}$	Protein expression in yeast (complementation)
pMB1365	pRS415-Met25-Dbp7-GFP	Protein expression in yeast
pMB1380	pQE-80-nHis10-ZZ-TEV-Dbp7$_{DQGD}$	Recombinant protein expression
pMB1381	pRS415_ DBP7$_{DQGD_500updown}$	Protein expression in yeast (complementation)
pML104	pRSII426-Cas9	CRISPR-Cas genome editing

Supplementary Table 5. Yeast strains used in this study.

Name	Genotype	Reference
YMB006/BY4741a	MATa;hisΔ1;leu2Δ0;met15Δ0;uraΔ0	Euroscarf
YMB489	YMB006; *dbp7*-HisTAP (URA3)	D. Tollervey
YMB1470	YMB006; *dbp7*::kanMX6	Euroscarf
YMB1479	BY4741a; *ssf1*-TAP (HIS3); pMB1365 (LEU2)	Open Bios.; This study

YMB1480	BY4741a; *arx1*-TAP (HIS3); pMB1365 (LEU2)	Open Bios.; This study
YMB1481	BY4741a; *nip7*-TAP (HIS3); pMB1365 (LEU2)	Open Bios.; This study
YMB1482	BY4741a; *npa1*-TAP (HIS3); pMB1365 (LEU2)	Euroscarf; This study
YMB1483	BY4741a; *nop2*-HisTAP (HIS3); pGAL-3xHA-*SPB*4 (kanMX6); pMB1365 (LEU2)	Bohnsack lab; This study
YMB1484	YMB006; $pGAL_S$-3xHA-*prp43* (natNT2); *rpf2*-HisTAP (HIS3); pMB1365 (LEU2)	Bohnsack lab; This study
YMB1485	YMB006; *erb1*-HisTAP (HIS3); pMB1365 (LEU2)	This study
YMB1487	YMB006; pMB031 (LEU2)	This study
YMB1488	YMB1470; pMB031 (LEU2)	This study
YMB1489	YMB1470; pMB1364 (LEU2)	This study
YMB1490	YMB1470; pMB1381 (LEU2)	This study
YMB1533	YMB006; *dbp7*-3xHA (HIS3)	This study
YMB1541	YMB006; $pGAL_S$-3xHA-*dbp7 (n*atNT2)	This study
YMB1693	YMB006; *dbp7*-TAP (kanMX6)	This study
YMB1819	YMB006; *nop2*-TAP (URA3)	This study
YMB1825	YMB1541; *nop2*-TAP (URA3)	This study
YMB1829	YMB1541; *npa2*-TAP (URA3)	This study
YMB1831	YMB1470; *nop2*-TAP (URA3)	This study
YMB1926	YMB1819; *cic1-6*xHA: (hphNT1)	This study
YMB1937	YMB1470; *cic1-6*xHA (hphNT1)	This study
YMB1945	YMB1819; pMB031 (LEU2)	This study
YMB1946	YMB1831; pMB031 (LEU2)	This study
YMB1947	YMB1831; pMB1364 (LEU2)	This study
YMB1948	YMB1831; pMB1381 (LEU2)	This study
YMB1951	YMB006; *dbp7*-yeGFP (hphNT1)	This study
YMB1954	YMB1951; pMB541 (LEU2)	This study
YMB1968	YMB006; pMB1364 (LEU2)	This study
YMB1969	YMB006; pMB1381 (LEU2)	This study
YMB1970	YMB1926; pMB31 (LEU2)	This study
YMB1972	YMB1937; pMB1364 (LEU2)	This study
YMB1974	YMB006; *snr34*::natNT2	This study
YMB1976	YMB006; *snr46*::natNT2	This study
YMB1978	YMB1470; *snr34*::natNT2	This study
YMB1979	YMB1470; *snr46*::natNT2	This study
YMB1981	YMB1937; pMB31 (LEU2)	This study
YMB1983	YMB1937; pMB1381 (LEU2)	This study
YMB1985	WT2	Jaafar et al.,
YMB1987	*snr190*mut	Jaafar et al.,
YMB1988	*dbp7*::kanMX6; *snr190*mut	Jaafar et al.,
YMB1989	*dbp7*::kanMX6; snr*190*wt	Jaafar et al.,

Supplementary Table 6. Antibodies used in this study

Name	Source	Dilution
Anti-HA	Sigma cat # H3663	1:1000
Anti-Pgk1	ThermoFisher Scientific cat # 459250	1:7500
Anti-PAP	Sigma cat # P1291	1:5000

Anti-CBP	Antibodies-online cat # ABIN3181196	1:5000
Anti-GFP	Roche cat # 11814460001	1:1000
Anti-Rpl3	Mybio-source cat # MBS9214187	1:1000
Anti-Rpl15	Aviva systems bio cat # ARP65141_P050	1:1000
Anti-Rps14	Aviva systems bio cat # ARP40322_T100	1:1000

Supplementary Methods

Fluorescence microscopy

Exponentially growing cells in synthetic media without leucine were imaged at room temperature automatically with a Nikon Ti2 2-E inverted microscope. Focal plane was set using Perfect Focus System (Nikon). Images were acquired using an oil objective lens (Plan Apo Lambda 100x/1.45 oil) at 100x magnification using the appropriate filters for GFP (Ex 470/24, Em 520/35, Dichroic 488) or mRFP (Ex 395/25, Em433/24, Dichroic 405).

Detection of pseudouridine by primer extension

10 µl whole cell RNA (10 µg) was added to 50 µl BEU buffer (7 M urea, 4 mM EDTA, 50 mM Bicine, pH 8.5) supplemented with or without 0.2 M N-cyclohexyl-N′-β-(4-methylmorpholinium) ethylcarbodiimide (CMC) following by incubation at 37 °C for 1 h. The CMC treated or untreated RNA (+/- CMC) was ethanol precipitated in the presence of 0.3 M KOAc and 15 µg glycogen. The pelleted and dried RNA was resuspended in alkaline buffer (50 mM Na_2CO_3, 2 mM EDTA, pH 10.4) and incubated at 37 °C for 2 h to preferentially removing CMC from G and U but not pseudouridine (Ψ). The alkaline treated RNA was PCI (Phenol:Chloroform:Isoamyl Alcohol (25:24:1, v/v), pH 6.7) extracted, ethanol precipitated and the pelleted RNA was washed with 70% ethanol, dried and resuspended in RNase free H_2O. 250 ng of denatured and treated or untreated RNA (+/- CMC) was reverse transcribed using 5 U AMV (Promega) and 0.5 pmol 5' [^{32}P]-labeled DNA oligonucleotide (Supplementary Table 4) in 1x AMV buffer supplemented with 0.1 M DTT and 1 mM dNTP at 42 °C for 30 min. The cDNA was subsequently treated with 5 U RNase H at 37 °C for 20 min, ethanol precipitated and resuspended in 1x UBB loading buffer (25 % urea, 5 mM EDTA, 0.5 mg/mL bromophenol blue, 0.5 mg/ml xylene cyanol). Reactions were separated on a denaturing (urea) 10% polyacrylamide gel together with a sequencing ladder that was generated by sequencing of a PCR product spanning the rRNA sequence of interest using the Thermo Sequenase Cycle Sequencing Kit (Thermo Scientific) and the same 5' [^{32}P]-labeled DNA oligonucleotide as in

the corresponding primer extension reaction. The gel was dried, exposed to a Phosphorimager screen and scanned on a Typhoon scanner (Amersham). Gel images were analyzed using Fiji software.

Verification of genomic alterations in yeast strains

1.5 ml of saturated yeast culture were harvested, resuspended in 250 μL of Lysis Buffer (2% Triton-X-100, 100 mM NaCl, 10 mM Tris-HCl pH 8, 1 mM EDTA, 1% SDS) and 250 μL PCI in the presence of glass beads. Cells were lysed by vortexing at 4°C for 5 min and after centrifugation, DNA in the upper aqueous phase was ethanol precipitated. Genomic DNA served as a temple for PCR reactions using primers (Supplementary Table 4) and Taq polymerase under appropriate conditions.

DMS chemical probing

Dimethyl sulphate (DMS) structure probing[3-5] was performed to analyze nucleotide accessibility of ITS2 nucleotides. Briefly, complexes associated with Nop2-TAP were immobilized on IgG sepharose as described in the main text and were treated or not treated with DMS (final concentration 0.5%) for 2 min followed by quenching by addition of 250 mM β-mercaptoethanol. Co-precipitated RNAs were extracted and used as templates for primer extension reaction using 5'-[^{32}P]-labelled DNA oligonucleotides (5'-CGCTCTCTTCTTATCGATAACG-3' and 5'- GCACGCAGAGAAACCTCTCTTTGG-3') and SuperScript III reverse transcriptase. cDNA fragments were separated in a 10% denaturing (7 M Urea) PAGE alongside a sequencing ladder generated by primer extension on total RNA in the presence of dideoxynucleotides. Signals were visualized using a phosphorimager.

Color-coded mapping of CRAC data onto rRNA secondary structures

The numbers of sequencing reads with the Dbp7-HTP CRAC dataset mapping to each nucleotide of the 25S rRNA according to the pyPileup output was represented in a color code

on the secondary structure of the *S. cerevisiae* LSU rRNA available at http://apollo.chemistry.gatech.edu/RibosomeGallery/eukarya/S%20cerevisiae/index.html[6] using the following Python script.

```
import csv
import os, sys
from lxml import etree
import re
from optparse import OptionParser

parser = OptionParser()

parser.add_option("-f", "--File", dest="Input_File",help="pyPileup.py-file", metavar="FILE",default=None)
parser.add_option("-S", "--SVG_SSU", dest="SVG_SSU",help="Input file of SVG-SSU",
metavar="FILE",default=None)
parser.add_option("-L", "--SVG_LSU", dest="SVG_LSU",help="Input file of SVG-LSU",
metavar="FILE",default=None)
parser.add_option("-o", "--Output_Folder", dest="Output_Folder",help="Output_Folder",
metavar="FILE",default=None)
parser.add_option("-n", "--Name", dest="Name",help="specify sample name", metavar="ProteinXY
(TEXT)",default="Sample")
parser.add_option("-c", "--Cutoff", dest="Cutoff",help="Minimum number of hits that will be colored on 2D-
structure", metavar="Number (INTEGER)",default=10)
parser.add_option("-x", "--Cutoff_X_links", dest="Cutoff_Crl",help="Minimum number of X-Links that will be
colored on 2D-structure", metavar="Number (INTEGER)",default=5)
parser.add_option("-d", "--delete_Data", dest="Del_old_Data",help="Delete colors of data stored in the SVG-file ",
metavar=" 1 = delete, 0 = do not modify",default=1)

(options, args) = parser.parse_args()
status = sys.stdout

Input_File = options.Input_File
SVG_SSU = options.SVG_SSU
SVG_LSU = options.SVG_LSU
Output_Folder = options.Output_Folder
Name = str(options.Name)
Cutoff = int(options.Cutoff)
Cutoff_Crl = int(options.Cutoff_Crl)
Organism = str("Sc")
Del_old_Data = str(options.Del_old_Data)


status.write('\n' )
status.write("pyPileup_2_2DStructure:" + '\n' )
status.write("Input_File: " + str(Input_File) +'\n' )
status.write("SVG_SSU: " +str(SVG_SSU) +'\n' )
status.write("SVG_LSU: " +str(SVG_LSU) +'\n' )
status.write("Output_Folder: " +str(Output_Folder) +'\n' )
status.write("Sample Name: " +str(Name) +'\n' )
status.write("Cutoff: " +str(Cutoff) +'\n' )
status.write("Cutoff-X-Links: " +str(Cutoff_Crl) +'\n' )
status.write('\n' )

if not Input_File:
    parser.error("File Missing: Specify Input_File" + '\n' )

    parser.error("File Missing: Specify SVG File for SSU (http://apollo.chemistry.gatech.edu/RibosomeGallery/)" +
'\n' )
if not SVG_LSU:
    parser.error("File Missing: Specify SVG File for SSU (http://apollo.chemistry.gatech.edu/RibosomeGallery/)" +
'\n')
if not Output_Folder:
    parser.error("Folder Missing: Specify Output Folder" + '\n')
```

```
########   Input  #########

#Organism = "Sc"
#Name = "Test"
#Cutoff = 10
#Del_old_Data = 1

########################

if Organism == "Sc":
    Length_SSU = 1800
    Length_LSU = 3675
    End_58S = 158
    End_25S = 3554
    End_5S = 3675

# Handle pyCRAC File
Maxlist = []
Maxlist_Crl = []
Data = []
content = open(Input_File, 'rU').read()
blocks = content.split('\n\n')
for block in blocks:
    lines = block.split('\n')
    for line in lines:
        line.rstrip('\n')
        Row = line.split('\t')
        if str(Row[0]) == str('# total number of mapped reads:'):
            Mapped_Reads = float(Row[1])
        if str(Row[0]).startswith('RDN'):
            Annotation = str(Row[0])
            Pos = int(Row[1])
            Sequence = str(Row[2])
            Hit = float(Row[3])
            Subs = float(Row[4])
            Del = float(Row[5])
            Crosslinks = Subs + Del
            Line = (Annotation, Pos, Hit, Crosslinks)
            Data.append(Line)

for i in Data:
    Maxlist.append(i[2])
    Maxlist_Crl.append(i[3])
Max_Val = max(Maxlist)
Max_Val_Crl = max(Maxlist_Crl)
#print Max_Val
#print Max_Val_Crl
RDN_Data = []
for i in Data:
    Norm_Hit = i[2]/Max_Val*100
    Norm_Hit = "%8.2f"%Norm_Hit
    Norm_Crl = i[3]/Max_Val_Crl*100
    Norm_Crl = "%8.2f"%Norm_Crl
    Line = (i[0], i[1], Norm_Hit, Norm_Crl)
    RDN_Data.append(Line)

# 2D mapping
def Mapping_2D(SVG_File, Data_Array, Output_File):

    NSMAP = {None: 'http://www.w3.org/2000/svg','inkscape': 'http://www.inkscape.org/namespaces/inkscape'}
    SVG_NS = "http://www.w3.org/2000/svg"       #SVG Namespaces

    Color_Gradient = ("BF2020","C32B1D","C8361B",
            "CC4119","D14C16","D65714",
            "DA6212","DF6D0F","E3780D",
            "E8830B","ED8E09","EE9916",
```

```
            "EFA423","F0B030","F1BB3D",
            "F2C74A","F3D257","F4DE64",
            "F5E971","F7F57E")

    def Step_Function(low,up,leng):
        list = []
        step = (up - low) / float(leng)
        for i in range(leng):
            list.append(low)
            low = low + step
        return list
    Datarange = 100 - Cutoff
    StepNumber = len(Color_Gradient)
    Steps = Datarange / float(StepNumber)
    Datasteparray = Step_Function(Cutoff, 100, StepNumber)
    #Define Color for Value
    def Gradient(Zahl):
        for i in Datasteparray:
            j = i+Steps
            global Color
            if float(Zahl) >= i and float(Zahl) < j:
                Index = Datasteparray.index(i)
                RevGrad = Color_Gradient[::-1]
                Color = RevGrad[Index]
            if float(Zahl) < Cutoff:
                Color = "AAAAAA"
        return Color

    #Create NEW-SVG-File
    New_SVG = open(Output_File, 'w')
    Style  = etree.Element('{http://www.w3.org/2000/svg}svg', nsmap=NSMAP)
    File = etree.parse(SVG_File)
    root = File.getroot()
    for l in root:
        Style.append(l)
    for i in Style:
        Item = i.items()
        ID_Style = str(i.get("id"))
        if ID_Style == "Labels_Text":
            print "ok"
            Data_Labels = etree.SubElement(Style, '{http://www.w3.org/2000/svg}g', id="Data_Labels")
            Name_Helicase = etree.SubElement(Data_Labels, '{http://www.w3.org/2000/svg}text', x='100', y='731.00',
fill='#221F1F', style='font-family:Myriad Pro;font-size:12px;text-anchor:left;dominant-baseline:top')
            Name_Helicase.text = str(Name)
            Cutoff_Helicase = etree.SubElement(Data_Labels, '{http://www.w3.org/2000/svg}text', x='100', y='744.00',
fill='#221F1F', style='font-family:Myriad Pro;font-size:10px;text-anchor:left;dominant-baseline:top')
            Cutoff_Helicase.text = "Cutoff: " + str(Cutoff)
            Cutoff_Crosslinks = etree.SubElement(Data_Labels, '{http://www.w3.org/2000/svg}text', x='100',
y='757.00', fill='#221F1F', style='font-family:Myriad Pro;font-size:10px;text-anchor:left;dominant-baseline:top')
            Cutoff_Crosslinks.text = "Cutoff-X-Links: " + str(Cutoff_Crl)
        if ID_Style == "Data1" or ID_Style == "Circles":
            print "yes"
            Content = i.getchildren()
            Length = len(Content)
            Index = Style.getchildren().index(i) + 1
            Data = etree.Element('{http://www.w3.org/2000/svg}g', id="Data")
            Style.insert(Index, Data)

            # Map Data
            linecount = []
            linespacing = []
            y_list = []
            x_list = []
            for k in Content:
                ID_1 = str(k.get("id"))
                CX = float(k.get("cx"))
                CY= float(k.get("cy"))
```

```
            if Del_old_Data == 1:
                k.set("fill", "#FFFFFF")
                k.set("stroke", "#FFFFFF")
            y_list.append(CY)
            x_list.append(CX)
        y_min = min(y_list)
        x_max = max(x_list) + 20
        for line in Data_Array:
            Count = int(line[1])
            nm = str(line[0])
            hit = float(line[2])
            crl = float(line[3])

            Color = Gradient(hit)
            Color_Crl = '1E90FF'

            if Length == int(Length_SSU):
                for j in range(Length_SSU):
                    ID = int(Content[j].get("id"))
                    CX = Content[j].get("cx")
                    CY = Content[j].get("cy")
                    if ID == Count and re.search('RDN18-2', nm):
                        Circle = etree.SubElement(Data, '{http://www.w3.org/2000/svg}circle', Nuclotide_ID=str(ID),
cx=str(CX), cy=str(CY), fill= "#"+str(Color), strokewidth="0.5", strokemiterlimit="10", r="2")
                    #if ID == Count and re.match('RDN18-2', nm) and Cutoff_Crl <= crl:
                        #Circle = etree.SubElement(Data, '{http://www.w3.org/2000/svg}circle', Nuclotide_ID=str(ID),
cx=str(CX), cy=str(CY), fill= "#"+str(Color_Crl), strokewidth="0.5", strokemiterlimit="10", r="2")
            if Length == int(Length_LSU):
                for j in range(End_58S):
                    ID = int(Content[j].get("id"))
                    CX = Content[j].get("cx")
                    CY= Content[j].get("cy")
                    if ID == Count and re.search('RDN58-2', nm):
                        Circle = etree.SubElement(Data, '{http://www.w3.org/2000/svg}circle', Nuclotide_ID=str(ID),
cx=str(CX), cy=str(CY), fill= "#"+str(Color), strokewidth="0.5", strokemiterlimit="10", r="2")
                    #if ID == Count and re.search('RDN58-2', nm) and Cutoff_Crl <= crl:
                        #Circle = etree.SubElement(Data, '{http://www.w3.org/2000/svg}circle', Nuclotide_ID=str(ID),
cx=str(CX), cy=str(CY), fill= "#"+str(Color_Crl), strokewidth="0.5", strokemiterlimit="10", r="2")
                for j in range(End_58S, End_25S):
                    ID = int(Content[j].get("id"))
                    CX = Content[j].get("cx")
                    CY= Content[j].get("cy")
                    if ID == Count and re.search('RDN25-2', nm):
                        Circle = etree.SubElement(Data, '{http://www.w3.org/2000/svg}circle', Nuclotide_ID=str(ID),
cx=str(CX), cy=str(CY), fill= "#"+str(Color), strokewidth="0.5", strokemiterlimit="10", r="2")
                    #if ID == Count and re.search('RDN25-2', nm) and Cutoff_Crl <= crl:
                        #Circle = etree.SubElement(Data, '{http://www.w3.org/2000/svg}circle', Nuclotide_ID=str(ID),
cx=str(CX), cy=str(CY), fill= "#"+str(Color_Crl), strokewidth="0.5", strokemiterlimit="10", r="2")
                for j in range(End_25S, End_5S):
                    ID = int(Content[j].get("id"))
                    CX = Content[j].get("cx")
                    CY= Content[j].get("cy")
                    if ID == Count and re.search('RDN5-2', nm):
                        Circle = etree.SubElement(Data, '{http://www.w3.org/2000/svg}circle', Nuclotide_ID=str(ID),
cx=str(CX), cy=str(CY), fill= "#"+str(Color), strokewidth="0.5", strokemiterlimit="10", r="2")
                    #if ID == Count and re.search('RDN5-2', nm) and Cutoff_Crl <= crl:
                        #Circle = etree.SubElement(Data, '{http://www.w3.org/2000/svg}circle', Nuclotide_ID=str(ID),
cx=str(CX), cy=str(CY), fill= "#"+str(Color_Crl), strokewidth="0.5", strokemiterlimit="10", r="2")


    New_SVG.write('<?xml version=\"1.0\" standalone=\"no\"?>\n')
    New_SVG.write('<!DOCTYPE svg PUBLIC \"-//W3C//DTD SVG 1.1//EN\"\n')
    New_SVG.write('\"http://www.w3.org/Graphics/SVG/1.1/DTD/svg11.dtd\">\n')
    SVG_Output = etree.tostring(Style, pretty_print=True)
    New_SVG.write(SVG_Output)
    New_SVG.close()
```

```
Mapping_2D(SVG_SSU, RDN_Data, Output_Folder + "2D_pyCRAC_18S_" + Name + "_" + str(Cutoff) + "pc" +
".svg")
Mapping_2D(SVG_LSU, RDN_Data, Output_Folder + "2D_pyCRAC_25S_" + Name + "_" + str(Cutoff) + "pc" +
".svg")

if __name__ == "__main__":
    pass
```

Color-coded mapping of CRAC data onto the tertiary structure of the 25S rRNA within PDB: 6EM1

The numbers of sequencing reads with the Dbp7-HTP CRAC dataset mapping to each nucleotide of the 25S rRNA was represented in a color code on the tertiary structure of the 25S rRNA within PBD: 6EM1[7] using the Python script given below. The mochi file generated by the pyCRAC pipeline served as input. The genome positions within the mochi file were converted into nucleotide positions on the rRNAs in three .txt files (25S_Yeast_Ribosome_Database.txt (genome 455182 = 25S rRNA 1, genome 451787 = 25S rRNA 3396), 58S_Yeast_Ribosome_Database.txt (genome 464709 = 5.8S rRNA 1, genome 464552 = 5.8S rRNA 158), ITS2_Yeast_Ribosome_Database.txt (genome 464551 = ITS2 1, genome 464320 = ITS2 232)).

```
import __main__
__main__.pymol_argv = [ '/sw/lib/pymol-py27/bin/pymol', '-c'] #no GUI
from pymol import cmd,stored
import pymol
from time import sleep
import csv
import os, sys
from lxml import etree
import re
from optparse import OptionParser

parser = OptionParser(usage="%prog [-M] [-p] [-T] [-o] ")

parser.add_option("-M", "--Mochi", dest="Mochi",help="Folder containing Mochifiles with CRAC hits",
metavar="Folder",default=None)
parser.add_option("-p","--Database",dest="Databasepath",help="Database folder with Tab delimited file
containing chromosomal position (Mochi) and  rRNA position in PDB chain",metavar="Folder",default=None)
parser.add_option("-T","--Template_File",dest="template_file",help="PDB file to include CRAC
hits",metavar="File",default=None)
parser.add_option("-o","--output_file",dest="output_file",help="otuput folder
folder",metavar="Folder",default=None)

(options, args) = parser.parse_args()
status = sys.stdout

Mochi = options.Mochi
inpath2 = options.Databasepath
template_file = options.template_file
output_file =  options.output_file
```

```
if not Mochi:
    parser.error("File Missing: Specify folder containing Mochi files" + '\n' )

if not inpath2:
    parser.error("Files Missing:Tab delimited file with chromosomal position (Mochi) and corresponding position in
CIF file " + '\n')
if not output_file:
    parser.error("Folder Missing: Specify Output Folder" + '\n')
if not template_file:
    parser.error("CIF file template 6em1.cif path" + '\n')



def beevalues(mochifile,inpath2):
        name = mochifile.split('/')[-1].split('_')[0]
        # Create Global Variable for MaxValues and make a dictionary
        Max_ListLSU = []
        with open(mochifile,'rU') as mochiFile:
            mochiFile.next()
            chr17list = []
            for line_Max in mochiFile:
                if line_Max.startswith('chr12'):
                    Columnlist_Max = line_Max.split("\t")
                    mochicol2=int(Columnlist_Max[1])
                    mochistrand = str(Columnlist_Max[3])
                    if mochistrand == '-':
                        mochiHits=abs(int(Columnlist_Max[6]))
                        if (mochicol2>=464552 and mochicol2<=464709) or (mochicol2>=451787 and
mochicol2<=455182) or (mochicol2>=464320 and mochicol2<=464551):
                            Max_ListLSU.append(mochiHits)
                            chr17list.append((mochicol2,mochiHits))

            chr17_dic=dict(chr17list)
            Max_ValLSU = max(Max_ListLSU)
            print 'Max LSU Hit: {}'.format(Max_ValLSU)
        # Load the Database file into reader
        with open('{}25S_Yeast_Ribosome_Database.txt'.format(inpath2),'rU') as Database25S,\
            open('{}58S_Yeast_Ribosome_Database.txt'.format(inpath2),'rU') as Database58S,\
            open('{}ITS2_Yeast_Ribosome_Database.txt'.format(inpath2),'rU') as DatabaseITS2:
        # Create Output File: Goes consecutively through the rRNA Databases and searches for matches in the
dictionaries.
            Outlist=[]
            for line in Database25S:
                    line.strip('\n\r')
                    Position = line.split("\t")
                    Position_1 = int(Position[0])
                    if Position_1 in chr17_dic:
                        Hits=float(chr17_dic[Position_1])
                        Percent = (Hits / Max_ValLSU)*100
                        Outlist.append(('{0:.2f}'.format(Percent),'25S'))
                    else:
                        Outlist.append(('0.00','25S'))
            for line in Database58S:
                    Position = line.split("\t")
                    Position_1 = int(Position[0])
                    if Position_1 in chr17_dic:
                        Hits=float(chr17_dic[Position_1])
                        Percent = (Hits / Max_ValLSU)*100
                        Outlist.append(('{0:.2f}'.format(Percent),'5.8S'))
                    else:
                        Outlist.append(('0.00','5.8S'))
            for line in DatabaseITS2:
                Position = line.split("\t")
                Position_1 = int(Position[0])
                if Position_1 in chr17_dic:
                    Hits=float(chr17_dic[Position_1])
                    Percent = (Hits / Max_ValLSU)*100
```

```
            Outlist.append(('{0:.2f}'.format(Percent),'ITS2'))
        else:
            Outlist.append(('0.00','ITS2'))
    return name,Max_ValLSU,Outlist

def ThreeDeeMapping(Mochi,output_file,inpath2,template_file):

  pymol.finish_launching()
  inpath = Mochi
  # Define data location
  outpath2= output_file
  Databasepath=inpath2 #Path to 25S 5.8S ITS2 Databasefiles

    # List all data in folder
  for (dirpath, dirnames, filenames) in os.walk(inpath):
      listing = filenames
      break
  if ".DS_Store" in listing:
    listing.remove(".DS_Store")
    print listing
    # process each file in folder
  for i in listing:
        cmd.reinitialize()
        print "current file is: {}".format(i)
        # Load local Structures
        cmd.load(template_file)
        # open the file of new values
        name,MaxLSU,bValues=beevalues(inpath+i,Databasepath) #needs path to mochi and path to
Databsefiles, returns name, MaxValue_SSU, MaxValue_LSU, and list two columns (b-Valule, rRNA name)
        print name
        stored.NameA=name
        # create the global, stored array
        cmd.create("25S", "c. 1")
        cmd.create ("5.8S", "c. 2")
        cmd.create ("ITS2", "c. 6")
        stored.newB_25S = []
        stored.newB_58S = []
        stored.newB_ITS2 = []
        # read the new B factors from file
        for line in bValues:
          if line[1]=='25S':
            stored.newB_25S.append(float(line[0]))
          elif line[1]=='5.8S':
            stored.newB_58S.append(float(line[0]))
          elif line[1]=='ITS2':
            stored.newB_ITS2.append(float(line[0]))
        #rotate Ribosome
        cmd.rotate("x", "110")
        cmd.rotate("y", "100")
        cmd.rotate("z", "326")
        cmd.viewport("1200,1200")
        #remove OHX Atoms from PDB
        #cmd.remove("HETATM")
        #clear out the old B Factors
        cmd.alter("all","b=0.0")
        #update the B Factors with new properties
        cmd.alter("25S and n. P", "b=stored.newB_25S.pop(0)")
        cmd.alter("5.8S and n. P", "b=stored.newB_58S.pop(0)")
        cmd.alter("ITS2 and n. P", "b=stored.newB_ITS2.pop(0)")
        #color the protein based on the new B Factors of the alpha carbons
        cmd.spectrum("b", "yellow_red", minimum=0, maximum=100)
        cmd.color("grey", "25S & b < 20")
        cmd.color("grey", "5.8S & b < 20")
        cmd.color("grey", "ITS2 & b < 20")
        #Set Label positions
        cmd.set ("label_position", "(3,3,3)")
        #Create Header
```

```
cmd.select('6em1')
cmd.pseudoatom("Header")
cmd.label("Header", "stored.NameA")
cmd.set("label_position", "(0,150,0)", "Header")
cmd.set ("label_color", "white", "Header")
# switch Cartoonview ON
cmd.show("cartoon")
cmd.unset("ignore_case")
cmd.set("transparency", "0.6")
#Color Proteins
cmd.hide ("everything", "c. A:z")
cmd.hide ("everything", "c. 3:5")

cmd.create("'L3'", "c. B")
cmd.label ("'L3' and resi 20 and n. CA", "'L3'")
cmd.create("'L4'", "c. C")
cmd.label ("'L4' and resi 20 and n. CA", "'L4'")
cmd.create("'L6'", "c. E")
cmd.label ("'L6' and resi 20 and n. CA", "'L6'")
cmd.create("'L7'", "c. F")
cmd.label ("'L7' and resi 20 and n. CA", "'L7'")
cmd.create("'L8'", "c. G")
cmd.label ("'L8' and resi 20 and n. CA", "'L8'")
cmd.create("'L9'", "c. H")
cmd.label ("'L9' and resi 20 and n. CA", "'L9'")
cmd.create("'L13'", "c. L")
cmd.label ("'L13' and resi 20 and n. CA", "'L13'")
cmd.create("'L14'", "c. M")
cmd.label ("'L14' and resi 20 and n. CA", "'L14'")
cmd.create("'L15'", "c. N")
cmd.label ("'L15' and resi 20 and n. CA", "'L15'")
cmd.create("'L16'", "c. O")
cmd.label ("'L16' and resi 20 and n. CA", "'L16'")
cmd.create("'L17'", "c. P")
cmd.label ("'L17' and resi 20 and n. CA", "'L17'")
cmd.create("'L18'", "c. Q")
cmd.label ("'L18' and resi 20 and n. CA", "'L18'")
cmd.create("'L20'", "c. S")
cmd.label ("'L20' and resi 20 and n. CA", "'L20'")
cmd.create("'L23'", "c. V")
cmd.label ("'L23' and resi 20 and n. CA", "'L23'")
cmd.create("'L26'", "c. Y")
cmd.label ("'L26' and resi 20 and n. CA", "'L26'")
cmd.create("'L32'", "c. e")
cmd.label ("'L32' and resi 20 and n. CA", "'L32'")
cmd.create("'L33'", "c. f")
cmd.label ("'L33' and resi 20 and n. CA", "'L33'")
cmd.create("'L35'", "c. h")
cmd.label ("'L35' and resi 20 and n. CA", "'L35'")
cmd.create("'L36'", "c. i")
cmd.label ("'L36' and resi 20 and n. CA", "'L36'")
cmd.create("'L37'", "c. j")
cmd.label ("'L37' and resi 20 and n. CA", "'L37'")

cmd.create("'Brx1'", "c. A")
cmd.label ("'Brx1' and resi 20 and n. CA", "'Brx1'")
cmd.create("'Has1'", "c. D")
cmd.label ("'Has1' and resi 20 and n. CA", "'Has1'")
cmd.create("'Ebp2'", "c. J")
cmd.label ("'Ebp2' and resi 20 and n. CA", "'Ebp2'")
cmd.create("'Cic1'", "c. K")
cmd.label ("'Cic1' and resi 20 and n. CA", "'Cic1'")
cmd.create("'Mrt4'", "c. W")
cmd.label ("'Mrt4' and resi 20 and n. CA", "'Mrt4'")
cmd.create("'Nog1'", "c. b")
cmd.label ("'Nog1' and resi 20 and n. CA", "'Nog1'")
cmd.create("'Erb1'", "c. m")
```

```
            cmd.label ("'Erb1' and resi 20 and n. CA", "'Erb1'")
            cmd.create("'Nop7'", "c. n")
            cmd.label ("'Nop7' and resi 20 and n. CA", "'Nop7'")
            cmd.create("'Nop15'", "c. o")
            cmd.label ("'Nop15' and resi 20 and n. CA", "'Nop15'")
            cmd.create("'Nsa2'", "c. r")
            cmd.label ("'Nsa2' and resi 20 and n. CA", "'Nsa2'")
            cmd.create("'Nug1'", "c. s")
            cmd.label ("'Nug1' and resi 20 and n. CA", "'Nug1'")
            cmd.create("'Rlp7'", "c. t")
            cmd.label ("'Rlp7' and resi 20 and n. CA", "'Rlp7'")
            cmd.create("'Rlp24'", "c. u")
            cmd.label ("'Rlp24' and resi 20 and n. CA", "'Rlp24'")
            cmd.create("'Nop16'", "c. v")
            cmd.label ("'Nop16' and resi 20 and n. CA", "'Nop16'")
            cmd.create("'Rpf1'", "c. x")
            cmd.label ("'Rpf1' and resi 20 and n. CA", "'Rpf1'")
            cmd.create("'Tif6'", "c. y")
            cmd.label ("'Tif6' and resi 20 and n. CA", "'Tif6'")
            cmd.create("'YBL028C'", "c. z")
            cmd.label ("'YBL028C' and resi 20 and n. CA", "'YBL028C'")
            cmd.create("'Mak16'", "c. 3")
            cmd.label ("'Mak16' and resi 20 and n. CA", "'Mak16'")
            cmd.create("'Rrp1'", "c. 4")
            cmd.label ("'Rrp1' and resi 20 and n. CA", "'Rrp1'")
            cmd.create("'Nsa1'", "c. 5")
            cmd.label ("'Nsa1' and resi 20 and n. CA", "'Nsa1'")

            cmd.set ("label_color", "palecyan", "c. A:z")
            cmd.show ("surface", "c. A:z")
            cmd.color ("palecyan", "c. A:z")

            cmd.set ("label_color", "palecyan", "c. 3:5")
            cmd.show ("surface", "c. 3:5")
            cmd.color ("palecyan", "c. 3:5")

            cmd.hide("lines"," 25S")
            cmd.hide("sticks"," 25S")
            cmd.hide("lines"," 5.8S")
            cmd.hide("sticks"," 5.8S")
            cmd.hide("lines"," ITS2")
            cmd.hide("sticks"," ITS2")
            cmd.set("cartoon_ring_mode","0")
            #cmd.set("cartoon_ring_finder",'1')
            cmd.set("cartoon_nucleic_acid_mode",'3')
            #cmd.set("cartoon_side_chain_helper")
            #cmd.set("cartoon_ring_transparency", "1")
            cmd.set("cartoon_ladder_mode", "0")
            #cmd.cartoon("oval")
            #cmd.set("cartoon_oval_width", "0.8")
            #cmd.cartoon("rect")
            cmd.cartoon("dumbbell")
            cmd.set("cartoon_dumbbell_width", '0.4')
            cmd.set("cartoon_dumbbell_radius", '0.4')
            cmd.rebuild()

            #Adjust Zoom
            cmd.zoom("all", '40')
            sleep(1.0) #(in seconds)
            cmd.save("{}LSU_Kater_6EM1_{}.pse".format(outpath2,name), "w")# Get out!
            sleep(4.0) #(in seconds)
            '''cmd.disable("all")
            cmd.enable("all")
            cmd.refresh()
    cmd.quit()
ThreeDeeMapping(Mochi,output_file,inpath2,template_file)
```

Supplementary References

1. Longtine, M.S. *et al.* Additional modules for versatile and economical PCR-based gene deletion and modification in Saccharomyces cerevisiae. *Yeast* **14**, 953-961 (1998).
2. Janke, C. *et al.* A versatile toolbox for PCR-based tagging of yeast genes: new fluorescent proteins, more markers and promoter substitution cassettes. *Yeast* **21**, 947-962 (2004).
3. Martin, R. *et al.* A pre-ribosomal RNA interaction network involving snoRNAs and the Rok1 helicase. *RNA* **20**, 1173–1182 (2014).
4. Brüning, L. *et al.* RNA helicases mediate structural transitions and compositional changes in pre-ribosomal complexes. *Nat. Commun.* **9**, 5383 (2018).
5. Swiatkowska, A. *et al.* Kinetic analysis of pre-ribosome structure in vivo. *RNA* **18**, 2187–2200 (2012).
6. Petrov, A. S. *et al.* Secondary structures of rRNAs from all three domains of life. *PLoS One* **9**, e88222 (2014).
7. Kater, L. *et al.* Visualizing the Assembly Pathway of Nucleolar Pre-60S Ribosomes. *Cell* **171**, 1599-1610.e14 (2017).
