# Supplementary material for: The RNA helicase Dbp7 promotes domain V/VI compaction and stabilization of inter-domain interactions during early 60S assembly
Source: Nat Commun. 2021 Oct 22;12:6152. doi: 10.1038/s41467-021-26208-9 (PMC8536713; doi:10.1038/s41467-021-26208-9)
Supplement: Supplementary file 2 — Reporting summary [file 41467_2021_26208_MOESM2_ESM.pdf]

nature research

Corresponding author(s): Markus T. Bohnsack
Last updated by author(s): Aug 27, 2021

Reporting Summary

Nature Research wishes to improve the reproducibility of the work that we publish. This form provides structure for consistency and transparency in reporting. For further information on Nature Research policies, see our Editorial Policies and the Editorial Policy Checklist.

Statistics

For all statistical analyses, confirm that the following items are present in the figure legend, table legend, main text, or Methods section.

n/a	Confirmed	
☐	☒	The exact sample size (*n*) for each experimental group/condition, given as a discrete number and unit of measurement
☐	☒	A statement on whether measurements were taken from distinct samples or whether the same sample was measured repeatedly
☐	☒	The statistical test(s) used AND whether they are one- or two-sided<br>*Only common tests should be described solely by name; describe more complex techniques in the Methods section.*
☒	☐	A description of all covariates tested
☒	☐	A description of any assumptions or corrections, such as tests of normality and adjustment for multiple comparisons
☐	☒	A full description of the statistical parameters including central tendency (e.g. means) or other basic estimates (e.g. regression coefficient) AND variation (e.g. standard deviation) or associated estimates of uncertainty (e.g. confidence intervals)
☐	☒	For null hypothesis testing, the test statistic (e.g. *F*, *t*, *r*) with confidence intervals, effect sizes, degrees of freedom and *P* value noted<br>*Give P values as exact values whenever suitable.*
☒	☐	For Bayesian analysis, information on the choice of priors and Markov chain Monte Carlo settings
☒	☐	For hierarchical and complex designs, identification of the appropriate level for tests and full reporting of outcomes
☒	☐	Estimates of effect sizes (e.g. Cohen's *d*, Pearson's *r*), indicating how they were calculated

Our web collection on statistics for biologists contains articles on many of the points above.

Software and code

Policy information about availability of computer code

Data collection	ImageQuant TL, version 8.2, image analysis software, Cytiva<br>ImageStudio Lite version 5.2.5
Data analysis	Flexbar version 2.7 (Dodt et al., 2012)<br>Bowtie2 version 2.2.4 (Langmead and Salzberg, 2012)<br>PyCRAC read counting, GitLab project ID 1049 (Webber et al., 2014)<br>Python version 2.7<br>PyMOL, version 1.8, molecular graphics software, Schrödinger, LLC<br>ImageQuant TL, version 8.2, image analysis software, Cytiva<br>ImageStudio Lite version XX<br>RMS data analysis (Birkedal et al., 2015)<br>MaxQuant version 1.6.5.0 (Cox et al., 2008)<br>Perseus version 1.6.2.3 (Tyanova et al., 2016)<br>Custom python scripts and associated files used for visualization of CRAC read numbers on secondary and tertiary structures of the S. cerevisiae ribosomal RNAs are available as Supplementary Data 1.

For manuscripts utilizing custom algorithms or software that are central to the research but not yet described in published literature, software must be made available to editors and reviewers. We strongly encourage code deposition in a community repository (e.g. GitHub). See the Nature Research guidelines for submitting code & software for further information.

Data

Policy information about availability of data

All manuscripts must include a data availability statement. This statement should provide the following information, where applicable:

- Accession codes, unique identifiers, or web links for publicly available datasets
- A list of figures that have associated raw data
- A description of any restrictions on data availability

DATA AVAILABILITY
The CRAC datasets and their analyses for Dbp7-HTP and the wild-type yeast control shown in Fig. 4 are deposited in Gene Expression Omnibus (GEO) database [http://www.ncbi.nlm.nih.gov/geo/] under the accession code GSE160734. Sequencing reads were mapped to the S. cerevisiae genome (https://www.yeastgenome.org/strain/s288c). The RMS datasets presented here in Fig. 2a,b are also deposited in the GEO data base under the accession code GSE161347. The mass spectrometry proteomics data underlying Fig. 1g have been deposited to the ProteomeXchange Consortium via the PRIDE partner repository [https://www.ebi.ac.uk/pride/] with the dataset identifier PXD022625. MS-MS spectra were searched against the UniProt S. cerevisiae database (https://www.uniprot.org/proteomes/UP000002311; downloaded on Feb 2019 with 9731 entries). Source data are provided with this paper.

Field-specific reporting

Please select the one below that is the best fit for your research. If you are not sure, read the appropriate sections before making your selection.

- [x] Life sciences
- [ ] Behavioural & social sciences
- [ ] Ecological, evolutionary & environmental sciences

For a reference copy of the document with all sections, see nature.com/documents/nr-reporting-summary-flat.pdf

Life sciences study design

All studies must disclose on these points even when the disclosure is negative.

Sample size	Sample size was determined for each set of experiments to reveal reproducibility and/or statistical significance of the data. Statistical determination of sample size was not performed.
Data exclusions	No data were excluded from the analyses
Replication	Reproducibility was determined by replications and statistical analyses were appropriate.
Randomization	Yeast strains used in this study were selected at random from among those generated that contained the correct genotype. Randomization was not performed during biochemical and cell biological experiments.
Blinding	Blinded experiments were not performed in this study.

Reporting for specific materials, systems and methods

We require information from authors about some types of materials, experimental systems and methods used in many studies. Here, indicate whether each material, system or method listed is relevant to your study. If you are not sure if a list item applies to your research, read the appropriate section before selecting a response.

Materials & experimental systems

n/a	Involved in the study	
☐	☒	Antibodies
☐	☒	Eukaryotic cell lines
☒	☐	Palaeontology and archaeology
☒	☐	Animals and other organisms
☒	☐	Human research participants
☒	☐	Clinical data
☒	☐	Dual use research of concern

Methods

n/a	Involved in the study	
☒	☐	ChIP-seq
☒	☐	Flow cytometry
☒	☐	MRI-based neuroimaging

Antibodies

Antibodies used	Anti-HA (Sigma cat # H3663), Anti-Pgk1 (ThermoFisher Scientific cat # 459250), Anti-PAP (Sigma cat # P1291), Anti-CBP (Antibodies-online cat# ABIN3181196), Anti-Rpl3 (Mybio-source cat# MBS9214187), Anti-Rpl15 (Aviva systems bio cat# ARP65141_P050), Anti-Rps14 (Aviva systems bio cat#ARP40322_T100)
Validation	Antibodies were validated by western blotting. To validate antibodies against tags, extracts from wild type yeast or strains expressing tagged versions of specific proteins were analysed. For antibodies against ribosomal proteins, the detection of proteins of

appropriate sizes co-migrating with ribosomal subunits was determined by western blotting.

Eukaryotic cell lines

Policy information about cell lines

Cell line source(s)	All yeast strains used in this study are based on Saccharomyces cerevisiae BY4741 (S288C). The genotypes of all derived strains are given in Supplementary Table 5 associated with the manuscript.
Authentication	All strains were verified by (antibiotic) marker selection, western blotting and/or PCR amplification of specific genomic regions. All plasmids introduced were verified by Sanger sequencing.
Mycoplasma contamination	n/a
Commonly misidentified lines (See ICLAC register)	n/a
